# Production of borneol, camphor, and bornyl acetate using engineered *Saccharomyces cerevisiae*

**DOI:** 10.1016/j.mec.2025.e00259

**Published:** 2025-03-31

**Authors:** Masahiro Tominaga, Kazuma Kawakami, Hiro Ogawa, Tomomi Nakamura, Akihiko Kondo, Jun Ishii

**Affiliations:** a Engineering Biology Research Center, Kobe University, 1–1 Rokkodai, Nada, Kobe, 657–8501, Japan; b Graduate School of Science, Technology and Innovation, Kobe University, 1–1 Rokkodai, Nada, Kobe, 657–8501, Japan; c Department of Chemical Science and Engineering, Faculty of Engineering, Kobe University, 1–1 Rokkodai, Nada, Kobe, 657–8501, Japan; d Center for Sustainable Resource Science, RIKEN, 1–7–22 Suehiro, Tsurumi, Yokohama, 230–0045, Japan

**Keywords:** Monoterpene, Borneol, Camphor, Bornyl acetate, Yeast

## Abstract

Microbial production of bicyclic monoterpenes is of great interest because their production primarily utilizes non-sustainable resources. Here, we report an engineered *Saccharomyces cerevisiae* yeast that produces bicyclic monoterpenes, including borneol, camphor, and bornyl acetate. The engineered yeast expresses a bornyl pyrophosphatase synthase from *Salvia officinalis* fused with mutated farnesyl pyrophosphate synthase from *S*. *cerevisiae* and two mevalonate pathway enzymes (an acetoacetyl-CoA thiolase/hydroxymethylglutaryl-CoA [HMG-CoA] reductase and an HMG-CoA synthase) from *Enterococcus faecalis*. The yeast produced up to 23.0 mg/L of borneol in shake-flask fermentation. By additionally expressing borneol dehydrogenase from *Pseudomonas* sp. TCU-HL1 or bornyl acetyltransferase from *Wurfbainia villosa*, the engineered yeast produced 23.5 mg/L of camphor and 21.1 mg/L of bornyl acetate, respectively. This is the first report of heterologous production of camphor and bornyl acetate.

## Highlights

• Engineered yeasts producing borneol, camphor, and bornyl acetate were constructed.
• Camphor and bornyl acetate were produced heterogeneously for the first time.
• Two-phase fermentation increased these bicyclic monoterpene productions.

## 1 Introduction

Bicyclic monoterpenoids, including borneol, camphor, and bornyl acetate, are used in traditional herbal medicine by leveraging their biological activities, which include anti-inflammatory, analgesic, antibacterial, antitumor, and anti-anxiety effects, and they are also used in fragrances and cosmetics (Ma et al., 2023). These monoterpenes are commercially available and can be extracted with high enantioselectivity from natural sources (e.g., *Cinnamomum burmanni* and *Blumea balsamifera* for borneol [Li et al., 2022], *Cinnamomum camphora* for camphor [Zhou and Yan, 2016], and *Amomum villosum*, *Inula graveolens*, and *Tetraclinis articulata* for bornyl acetate [Zhao et al., 2023]). However, the supply chains of these products are unstable due to limited space for plant cultivation and low yield. Although racemic borneol and camphor can be chemically synthesized from α-pinene, a major constituent of turpentine oil (Ponomarev and Mettee, 2016), this process produces a toxic by-product, isoborneol, which may causez1 serious side effects. Furthermore, chemical synthesis of these compounds uses harmful catalysts, such as heavy metals (Ponomarev and Mettee, 2016). Thus, alternative methods to sustainably produce these monoterpenes are needed.

**Fig. 1:**
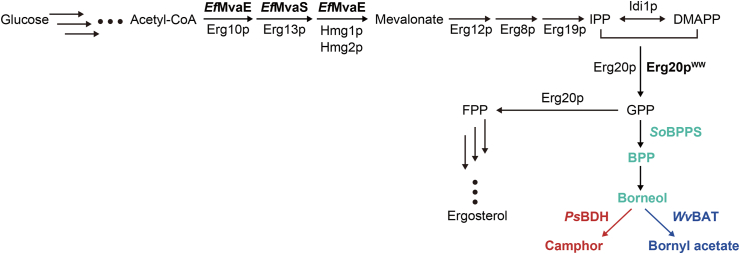
**Biosynthetic pathway of borneol, camphor, and bornyl acetate reconstituted in the yeast *S*. *cerevisiae*.** Heterologous enzymes are indicated in bold type.

Borneol, camphor, and bornyl acetate are naturally biosynthesized in the plants via the isoprenoid pathway, as follows (Fig. 1). Isoprenyl-pyrophosphate (IPP) and dimethylallyl pyrophosphate (DMAPP) are produced via the mevalonate pathway and condensed into geranyl-pyrophosphate (GPP) by farnesyl pyrophosphate synthetase, Erg20p. GPP is then further circularized to bornyl-pyrophosphate (BPP) by BPP synthase (BPPS), and BPP is dephosphorylated into borneol. Borneol dehydrogenase (BDH) and bornyl acetyltransferase (BAT) convert the resultant borneol into camphor and bornyl acetate, respectively.

BPPSs are found in a diverse array of plants, including *B. balsamifera*, *C. burmanni*, *A. villosum*, *Lavandula angustifolia*, *Salvia officinalis*, and *Lippia dulcis* (Despinasse et al., 2017; Wang et al., 2018; Ma et al., 2022). BDHs are found in plants (*C. camphora* [*L*.] *Presl*) (Ma et al., 2021) and bacteria (*Pseudomonas* sp. strain TCU-HL1) (Tsang et al., 2016). More recently, a plant BAT was identified for the first time in *Wurfbainia villosa* (homotypic synonym, *A. villosum*) (Liang et al., 2022). Microbial production of borneol by heterologous expression of plant BPPSs has also been reported. An engineered *Escherichia coli* strain reportedly produced 87.20 mg/L borneol in shake-flask fermentation (Lei et al., 2021), whereas an engineered strain of the yeast *Saccharomyces cerevisiae* produced 12.68 mg/L and 148.59 mg/L borneol in a shake flask and 5-L bioreactor, respectively (Ma et al., 2022). However, borneol productivity remains far below the level needed for practical use, and microbial production of camphor or bornyl acetate has not been reported to date. To establish a microbial platform to produce borneol, camphor, and bornyl acetate, we metabolically engineered the yeast *S*. *cerevisiae*, including engineering the heterologous expression of mevalonate pathway enzymes and a fusion enzyme between BPPS and an Erg20p mutant, with and without BDH or BAT.

## 2 Materials and methods

### 2.1 Strains, plasmids, synthetic DNAs, and primers

All synthetic gene cassettes examined in the study are listed in Supplementary Table 1. All synthetic DNA fragments were purchased from GeneArt (Thermo Fisher Scientific, Waltham, MA, USA). The following parental plasmids were used for plasmid construction: pRS405red (Tominaga et al., 2021) and pATP403red (Tominaga et al., 2021). Plasmid sequences are shown in **Supplementary Note 1**. Schematic illustrations of the construction of yeast strains used in this study are shown in Supplementary Figs. 1–3.

### 2.2 Media and reagents

Synthetic complete (SC, 0.67 % yeast nitrogen base without amino acids [BD Biosciences, San Jose, CA, USA], 2 % d-glucose [Nacalai Tesque, Kyoto, Japan], 0.2 % amino acids complete mix without l-methionine, l-leucine, l-histidine, and uracil [Supplementary Table 2]) and yeast peptone dextrose (YPD, 1 % yeast extract [Nacalai Tesque], 2 % Bacto peptone [BD Biosciences], 2 % d-glucose) media were used for incubating yeast strains. To obtain solid media, 2 % agarose was added.

### 2.3 Shake-flask fermentation and monoterpenoid quantification

**Fig. 2:**
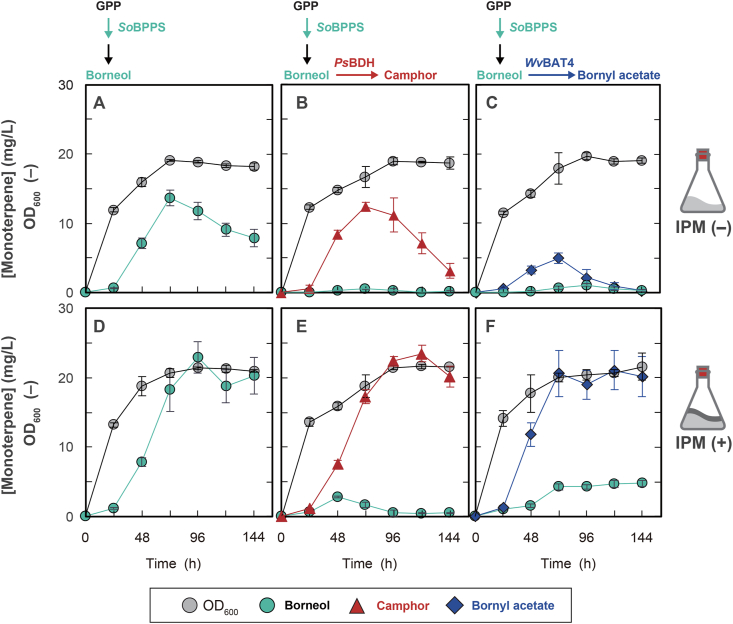
**Single- and two-phase shake-flask fermentation of yeasts to produce borneol, camphor, and bornyl acetate.** Yeast strains expressing Erg20p^WW^::*So*BPPS, *Ef*MvaE, and *Ef*MvaS (ScKZ045 [A, D]) or in further combination with *Ps*BDH (ScKZ048 [B, E]) or *Wv*BAT4 (ScKZ046 [C, F]). Yeasts were incubated in a shake flask without (A, B, C) or with (D, E, F) IPM overlay. Data are shown as the mean, and error bars show the SD of three independent experiments.

An overnight culture of yeast in 5 mL of SC (Fig. 2) or YPD (Supplementary Fig. 5) medium incubated at 30 °C at 150 rpm was transferred into 20 mL of YPD medium in a baffled shake flask (initial OD_600_ ∼0.1) and incubated at 30 °C at 200 rpm. At each time point, the OD_600_ was monitored using a UV-1280 UV–Vis spectrophotometer (Shimadzu, Kyoto, Japan), and the cell culture was vigorously mixed with an equivalent volume of ethyl acetate by vortexing for 10 min at 4 °C, followed by centrifugation for 10 min at 13,000 *g*. The levels of extracted borneol, camphor, and bornyl acetate in the organic phase were measured using a gas chromatography–mass spectrometry (GC-MS) system consisting of a GCMS-QP2010 Ultra (Shimadzu) spectrometer equipped with a DB-5MS column (0.25 mm × 30 m, membrane thickness of 0.25 μm; Agilent Technologies, Santa Clara, CA, USA) and AOC-20i auto-injector (Shimadzu), as described previously (Zhang et al., 2021) with the following modifications. Mass spectrometry was carried out in the SIM/Scan mode and *m/z* range of 50–200. Calibrations were performed using standards at a concentration of up to 50 μM with a coefficient of determination >0.99. The major quantifier ion for all three analytes was *m/z* 95. Other qualifier ions were as follows: *m/z* 67 and 110 for borneol, *m/z* 81 and 108 for camphor, and *m/z* 93 and 136 for bornyl acetate. For two-phase fermentation, isopropyl myristate (IPM) was added to the medium at a final concentration of 10 % (v/v), and cultivation was carried out by vortexing for 10 min at 4 °C. Finally, a 1-mL aliquot of the organic phase was used for analysis.

## 3 Results and discussion

To generate a yeast strain that produces borneol, the gene encoding BPPS without the N-terminal plastid-localization sequence (2–49 residues) from *S. officinalis* (*So*BPPS, GenBank: AAC26017.1) was codon-optimized for yeast (Supplementary Table 1) and fused to the mutated *ERG20* gene from yeast, which encodes mutant Erg20p with high GPP synthesis activity (Erg20p^F96W−N127W^, Erg20p^WW^), thereby enabling the efficient substrate channeling (Ignea et al., 2014) toward BPP. The resulting fusion gene was cloned into the yeast integration vector so that the BPPS gene could be expressed from the strong *TDH3* promoter. In addition, the genes encoding acetoacetyl-CoA thiolase/hydroxymethylglutaryl-CoA (HMG-CoA) reductase and HMG-CoA synthase from *Enterococcus faecalis* (*Ef*MvaE and *Ef*MvaS, respectively), which convert acetyl-CoA into mevalonate to produce more terpenoids in both *E*. *coli* (Tsuruta et al., 2009; Yoon et al., 2009) and the yeast *S*. *cerevisiae* (Peng et al., 2017), were also cloned into plasmid pATP403red (Tominaga et al., 2021). The resulting plasmids were successively integrated into the chromosome of the yeast strain ScKZ014 derived from strain BY4741 (Brachmann et al., 1998), which harbors a borneol-responsive transcription activator and reporter plasmid for the *in vivo* sensing of borneol (Supplementary Fig. 1 and Supplementary Note 1), thereby generating yeast strain ScKZ045 (Supplementary Fig. 2). When this strain was incubated in YPD medium, the concentration of borneol in the medium increased after 24 h and peaked at 72 h, reaching 13.6 mg/L before gradually decreasing (Fig. 2A and Supplementary Fig. 4). Note that the borneol concentration was >3-fold higher than that produced using the strain lacking expression of *Ef*MvaE and *Ef*MvaS (Supplementary Fig. 5).

To convert borneol into camphor and bornyl acetate, two strains were constructed that additionally express borneol dehydrogenase from *Pseudomonas* sp. TCU-HL1 (*Ps*BDH, GenBank: AOE86728.1) and bornyl acetyltransferase from *W. villosa* (*Wv*BAT4) (Liang et al., 2022). To do so, codon-optimized *PsBDH* and *WvBAT4* (Supplementary Table 1) were additionally cloned into the expression vectors for *Ef*MvaE and *Ef*MvaS and integrated into yeast strain ScKZ045, generating strains ScKZ048 and ScKZ046, respectively (Supplementary Fig. 3). These strains successfully produced camphor and bornyl acetate (Fig. 2B and C, and Supplementary Fig. 4) and exhibited the same time-course for borneol production. The concentrations of camphor and bornyl acetate reached 12.4 and 5.00 mg/L at 72 h, respectively, and then decreased. Borneol, camphor, and bornyl acetate are highly volatile. For instance, >20 % of borneol is lost from the medium after 48 h of incubation (Lei et al., 2021), which may contribute to the observed decrease in monoterpene production after 72 h.

To minimize the loss of volatile monoterpenes from the fermentation medium, we performed two-phase fermentation by adding IPM to the medium (Fig. 2D–F). IPM was added at a final concentration of 10 % (v/v), and cultivation was carried out by vortexing, followed by removal of an aliquot from the organic phase for analysis. As expected, the maximum concentrations of borneol, camphor, and bornyl acetate increased by 1.7-fold (23.0 mg/L at 96 h, Fig. 2D), 1.9-fold (23.5 mg/L at 120 h, Fig. 2E), and 4.2-fold (21.1 mg/L at 120 h, Fig. 2F), respectively. Note that the growth of all yeast strains was enhanced slightly by the addition of IMP, possibly because the toxic effects of the monoterpenes were reduced by extracting the compounds into the IPM phase (Brennan et al., 2012). Approximately 5 mg/L of borneol remained in the medium when producing bornyl acetate (Fig. 2F) but not when producing camphor (Fig. 2D), indicating that the enzymatic activity of *Wv*BAT4 is weaker than that of *Ps*BDH.

## 4 Conclusions

In this study, we established a microbial platform to produce borneol, camphor, and bornyl acetate using the yeast *S. cerevisiae*. To this end, we engineered yeast strains to express the rate-limiting enzymes (*Ef*MvaE and *Ef*MvaS) and the fusion enzyme of BPPS and Erg20p^WW^ with and without *Ps*BDH or *Wv*BAT4. This study is the first report of yeast production of camphor and bornyl acetate. Although the concentration of borneol produced was comparable to that of a previously reported yeast strain (Ma et al., 2022), overlaying IPM onto the medium improved borneol production by 1.7-fold, leading to the highest borneol titer in a shake flask reported for the yeast *S*. *cerevisiae*. Nevertheless, the borneol production was 4-fold lower than that reported for *E*. *coli* (Lei et al., 2021). Further improvement in production could be achieved by combining the modulation of competitive pathways (Paddon et al., 2013; Hull et al., 2014; Peng et al., 2017; Broker et al., 2018; Zhou et al., 2021; Tominaga et al., 2022; Wei et al., 2024), protein engineering to improve the enzymatic activity of BPPSs (Lei et al., 2021), amplification of the gene copy number of rate-limiting enzymes (Peng et al., 2022), and enzyme compartmentalization (Cheah et al., 2023). Evolutionary engineering for the production of borneol and camphor can be performed using recently developed genetically encoded biosensors that respond to borneol or camphor (Ikushima et al., 2015; Ikushima and Boeke, 2017; Tominaga et al., 2021; D'oelsnitz et al., 2022). Finally, a high-throughput platform could be developed to facilitate mutational analysis of various plant BDHs (Lin et al., 2023) and investigate their properties, such as product specificity (Hofer et al., 2021; Ma et al., 2021; Hu et al., 2022).

## CRediT authorship contribution statement

**Masahiro Tominaga:** Writing – review & editing, Writing – original draft, Visualization, Validation, Methodology, Investigation, Formal analysis, Data curation, Conceptualization. **Kazuma Kawakami:** Writing – original draft, Methodology, Investigation, Formal analysis, Data curation, Conceptualization. **Hiro Ogawa:** Methodology, Investigation, Formal analysis, Data curation. **Tomomi Nakamura:** Methodology, Formal analysis, Data curation. **Akihiko Kondo:** Supervision, Resources, Project administration, Conceptualization. **Jun Ishii:** Writing – review & editing, Supervision, Project administration, Funding acquisition, Conceptualization.

## Declaration of competing interest

The authors declare that they have no known competing financial interests or personal relationships that could have appeared to influence the work reported in this paper.

## Data Availability

Data will be made available on request.
