# Supplementary material for: Production of borneol, camphor, and bornyl acetate using engineered Saccharomyces cerevisiae
Source: Metab Eng Commun. 2025 Mar 31;20:e00259. doi: 10.1016/j.mec.2025.e00259 (PMC12002889; doi:10.1016/j.mec.2025.e00259)
Supplement: Multimedia component 1 [file mmc1.pdf]

Supplementary information for “Production of borneol, camphor, and bornyl acetate using engineered *Saccharomyces cerevisiae*”

- Supplementary Figs. 1-5
- Supplementary Tables 1 & 2
- Supplementary Note 1

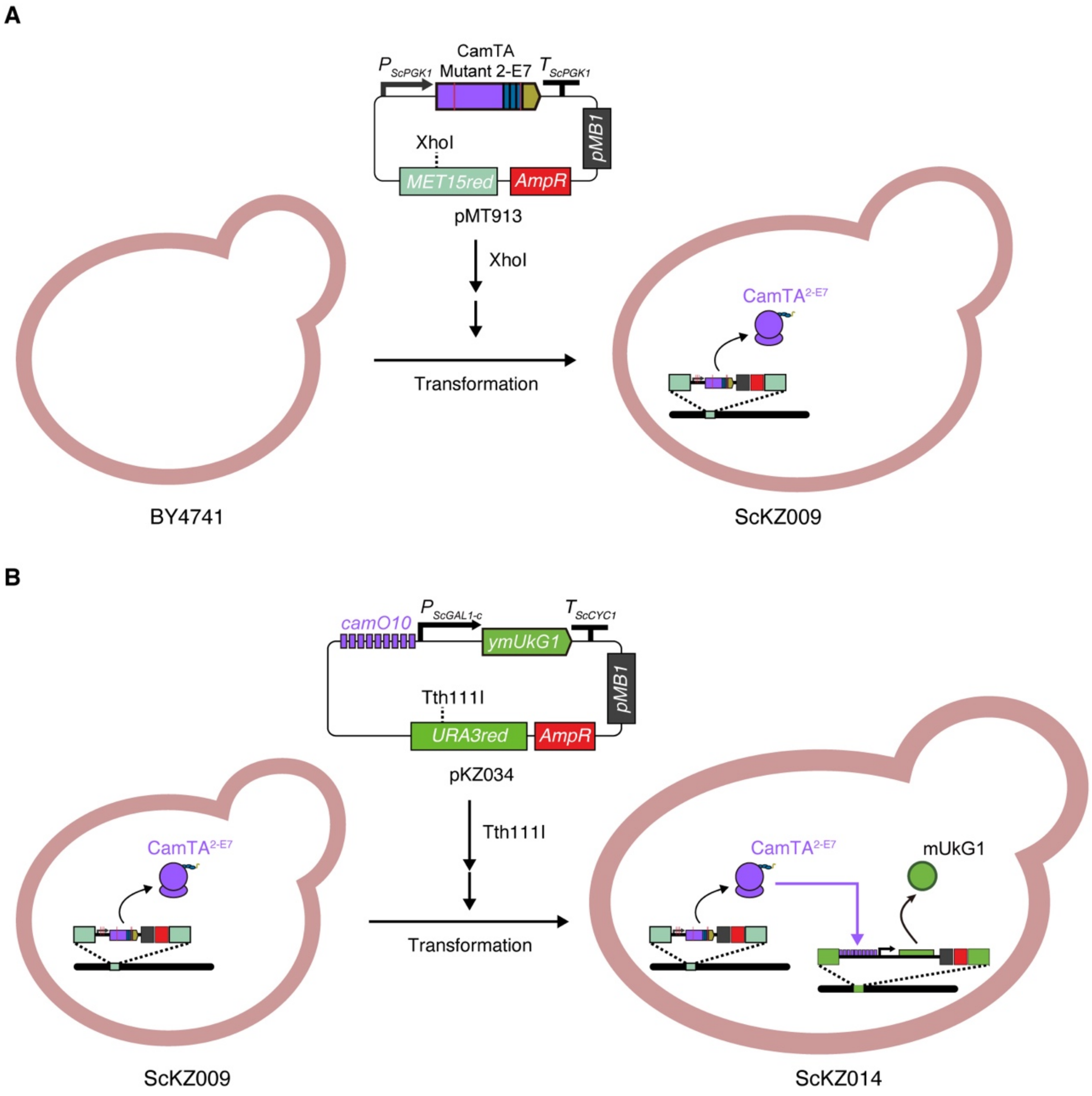

Supplementary Fig. 1. Schematic illustration of the construction of yeast strains harboring a borneol-responsive *in vivo* biosensor. A. First, the mutant expression vector for the synthetic transcription factor CamTA (2-E7) was chromosomally integrated into strain BY4741. **B**. The resulting strain (ScKZ009) was then transformed with the plasmid pKZ034 to generate strain ScKZ014.

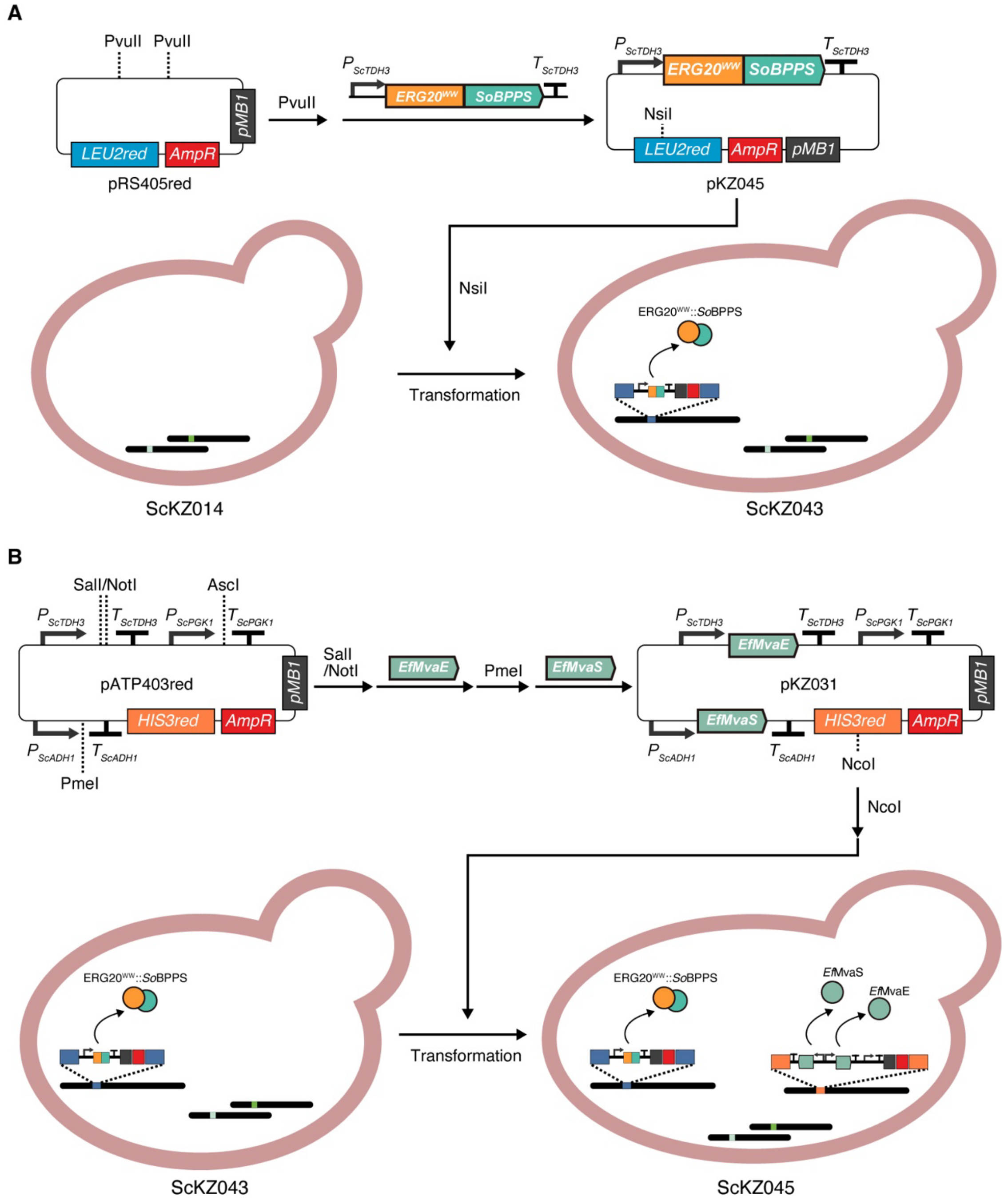

Supplementary Fig. 2. Sequential construction of yeast strain that simultaneously expresses Erg20p^{WW}::*So*BPPS, *Ef*MvaS, and *Ef*MvaE. A. A plasmid expressing Erg20p^{WW}::*So*BPPS (pKZ045) was integrated into strain ScKZ014 (**Supplementary Fig. 1**) to generate strain ScKZ043. **B**. A plasmid expressing *Ef*MvaS and *Ef*MvaE (pKZ031) was integrated into strain ScKZ014 to generate strain ScKZ045.

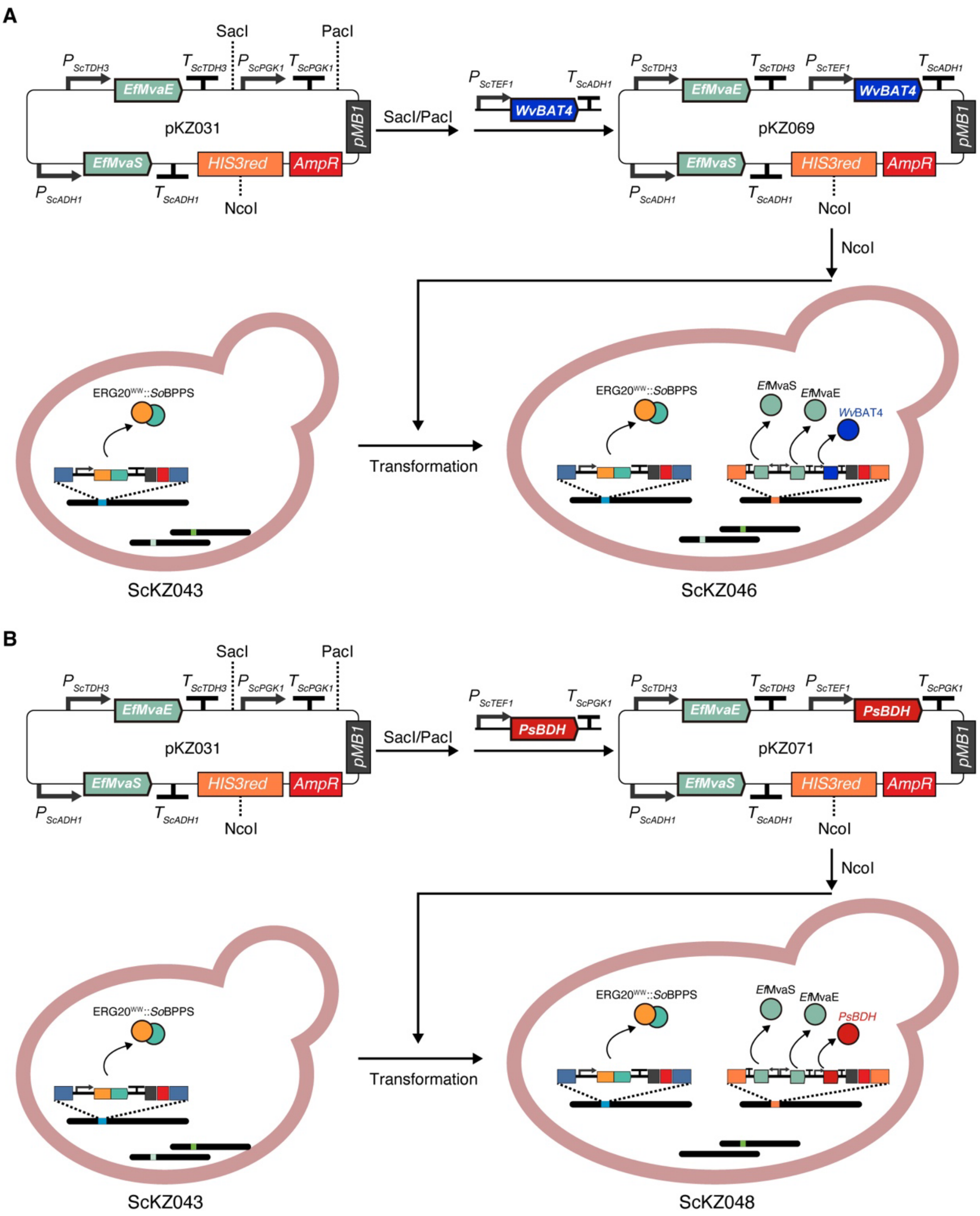

Supplementary Fig. 3. Construction of yeast strains that additionally express *Wv*BAT4 or *Ps*BDH. Yeast strain ScKZ043 (**Supplementary Fig. 2**) was transformed using a plasmid expressing *Ef*MvaS, *Ef*MvaE, and *Wv*BAT4 (pKZ059) (**A**) or *Ps*BDH (pKZ071) (**B**) to generate strains ScKZ046 and ScKZ048, respectively.

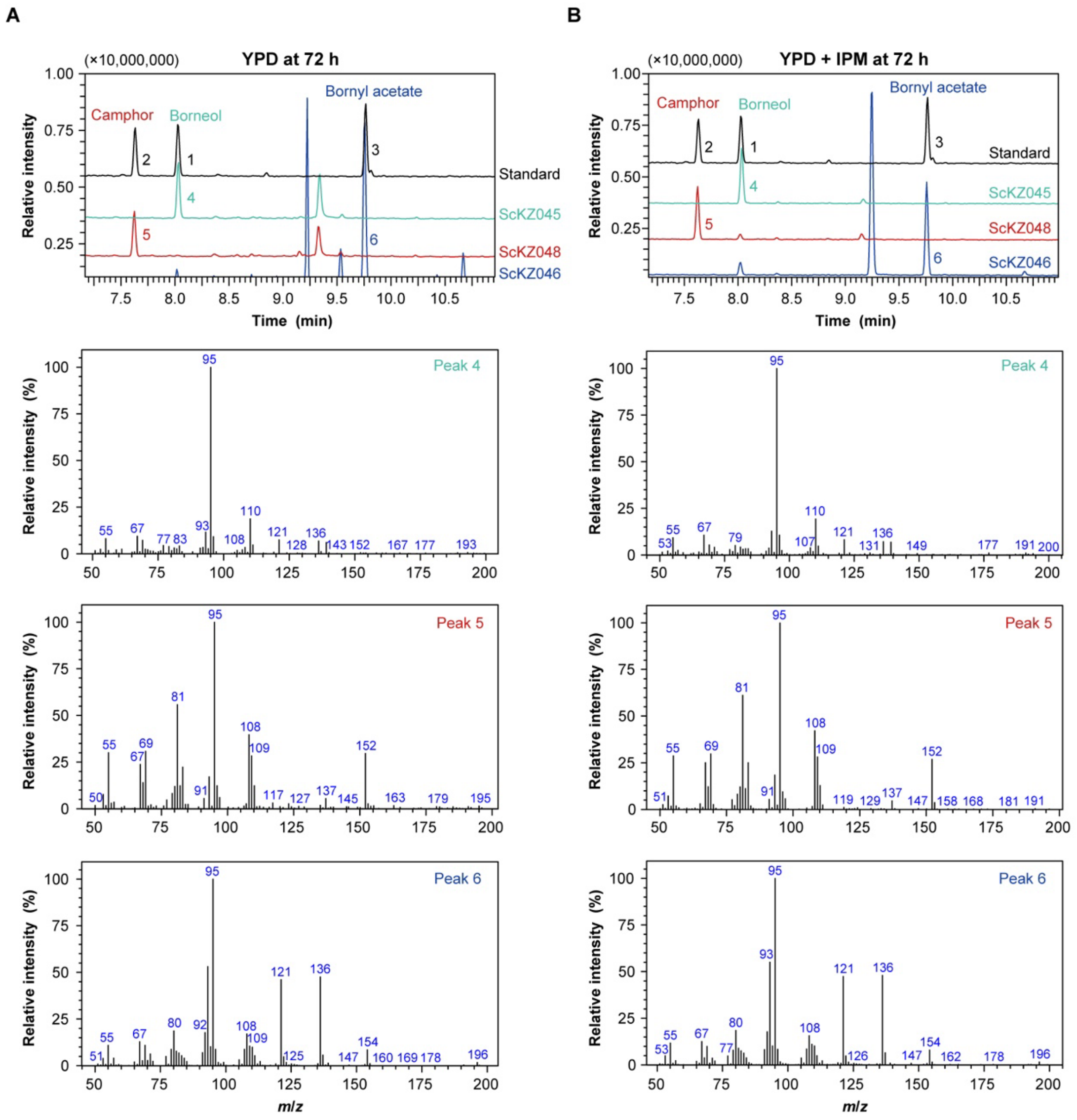

Supplementary Figure 4. Gas chromatography–mass spectrometry analysis of borneol, camphor, and bornyl acetate in extracts from engineered yeast strains (Fig. 2). Chromatograms of standard solution (1: borneol, 2: camphor; 3: bornyl acetate) and extracted products from yeast strains expressing Erg20p^{WW}::*So*BPPS, *Ef*MvaE, and *Ef*MvaS (ScKZ045), or in combination with *Ps*BDH (ScKZ048) or *Wv*BAT4 (ScKZ046), respectively, incubated for 72 h in YPD (**A**) or YPD+IPM (**B**) medium. Comparisons of mass spectra for peaks 4 through 6 are shown.

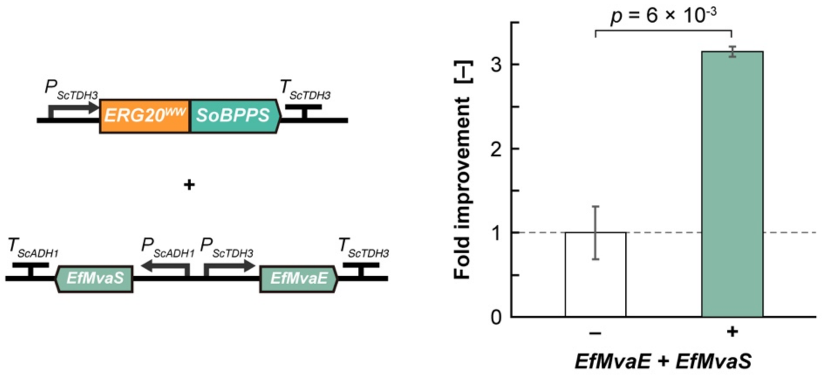

Supplementary Figure 5. Effect of *Ef*MvaE and *Ef*MvaS expression on borneol production in shake-flask. Yeast strains solely expressing Erg20p^{WW}::*So*BPPS (ScKZ043) or coexpressing with *Ef*MvaE and *Ef*MvaS (ScKZ045). Yeasts were incubated in a shake flask for 72 h. Data are shown as the mean, and error bars show the SD of three independent experiments.

Supplementary Table 1. Synthetic gene cassettes used in this study.

Cassette	Sequence	Note
P_{TDH3}-$ERG20^{WW}$ ::*SoBPPS*-T_{TDH3}	gaataaaaaacacgctttttcagttcgagtttatcattatcaatactgccatttcaaagaatacgtaaataa<br>ttaatagtagtgattttcctaactttatttagtcaaaaaattagccttttaattctgctgtaacccgtacatgc<br>ccaaaatagggggcgggttacacagaatatataacatcgtaggtgtctgggtgaacagtttattcctg<br>gcatccactaaatataatggagcccgctttttaagctggcatccagaaaaaaaaagaatcccagcacc<br>aaaatattgttttcttcaccaaccatcagttcataggtccattctcttagcgcaactacagagaacaggg<br>gcacaaacaggcaaaaaacgggcacaacctcaatggagtgatgcaacctgcctggagtaaatgatg<br>acacaaggcaattgacccacgcatgtatctatctcattttcttacaccttctattaccttctgctctctctgat<br>ttggaaaaagctgaaaaaaaaggttgaaaccagttccctgaaattattcccctacttgactaataagtat<br>ataaagacggtaggtattgattgtaattctgtaaatctatttcttaaacttcttaaattctacttttatagttagt<br>cttttttttagttttaaaacaccaagaacttagtttcgaataaacacacataaacaaacaaaatggcttcag<br>aaaaagaaattaggagagagagattcttgaacgttttccctaaattagtagaggaattgaacgcatcgc<br>ttttggcttacggtatgcctaaggaagcatgtgactggtatgcccactcattgaactacaacactccag<br>gcggtaagctaaatagaggtttgtccgttgtggacacgtatgctattctctccaacaagaccgttgaac<br>aattggggcaagaagaatacgaaaaggttgccattctaggttggtgcattgagttgttgcaggcttact<br>ggttggtcgccgatgatatgatggacaagtccattaccagaagaggccaaccatgttggtacaaggtt<br>cctgaagttggggaaattgccatctgggacgcattcatgttagaggctgctatctacaagcttttgaaat<br>ctcacttcagaaacgaaaaatactacatagatatcaccgaattgttccatgaggtcaccttccaaaccg<br>aattgggccaattgatggacttaatcactgcacctgaagacaaagtcgacttgagtaagttctccctaa<br>agaagcactccttcatagttactttcaagactgcttactattctttctacttgcctgtcgcattggccatgta<br>cgttgccggtatcacggatgaaaaggatttgaaacaagccagagatgtcttgattccattgggtgaata<br>cttccaaattcaagatgactacttagactgcttcggtaccccagaacagatcggtaagatcggtacaga<br>tatccaagataacaaatgttcttgggtaatcaacaaggcattggaacttgcttccgcagaacaaagaaa<br>gactttagacgaaaattacggtaagaaggactcagtcgcagaagccaaatgcaaaaagattttcaatg<br>acttgaaaattgaacagctataccacgaatatgaagagtctattgccaaggatttgaaggccaaaattt<br>ctcaggtcgatgagtctcgtggcttcaaagctgatgtcttaactgcgttcttgaacaaagtttacaagag<br>aagcaaaatggaagcccatcaaattagaaggtctggtaattatcaaccagccttgtgggattccaacta<br>cattcaatctttgaacactccatacaccgaagaaagacacttggatagaaaagccgaattgatcgttca<br>agtcaggatcttgttgaaagaaaagatggaaccagtccagcagttggaattgattcacgatttgaaata<br>cttgggcctgtccgatttttttccaggacgaaatcaaagaaatcttgggcgttatctacaacgaacataa<br>gtgcttccataacaacgaagttgagaagatggacttgtactttactgctttgggtttccgtttgttgagac<br>aacatggtttcaacatctcccaagatgttttcaactgcttcaagaacgaaaagggtattgacttcaaggc<br>ttctttggctcaagataccaaaggtatgttgcaattatacgaggccagtttcttgttgagaaagggtgaa<br>gatactttggaattggctagagaatttgctactaagtgcttgcagaaaaagttggatgaaggtggtaac<br>gaaatcgacgaaaatttgttgttgtggatcagacactctttggacttgccattgcattggagaatccaat	P_{TDH3}, $ERG20^{WW}$ ::*SoBPPS*, T_{TDH3}

	ctgttgaagctagatggttcattgatgcttatgctagaaggccagatatgaacccattgatttttgaattg<br>gccaagctgaacttcaacatcattcaagctactcaccagcaagaattgaaggatttgtcaagatggtg<br>gtccagattgtgttttccagaaaagttgccattcgtcagagatagattggtcgaatcttttttttgggccgt<br>tggtatgtttgaaccacatcaacacggttaccaaagaaaaatggctgctaccattatcgttttggccacc<br>gttattgatgacatctatgatgtttacggcaccttggatgaattggagttgtttactgataccttcaagaga<br>tgggataccgaatctattactagattgccctactacatgcaattgtgttattggggtgttcacaactacat<br>ctctgatgctgcttacgacatcttgaaagaacacggtttttctgcttgcagtacctgagaaaatccgttg<br>ttgatttggttgaagcctacttccatgaagctaagtggtatcattctggttacactccatctttggacgagt<br>atttgaacattgccaaaatctctgttgcttccccagctattatttctccaacttactttactttcgccaacgct<br>tctcatgataccgctgttattgattccttgtaccaataccacgacattttgtgtttggccggtattatcttga<br>gattgccagatgatttgggcacctcttactttgaattagcaagaggtgatgttccaaagaccattcagtg<br>ttacatgaaggaaactaacgcctctgaagaagaagctgttgaacacgttaagttcttgattagagaagc<br>ctggaaggatatgaacactgctattgctgctggttatccatttccagatggtatggttgctggtgctgcta<br>atattggtagagttgctcaattcatctacttgcatggtgatggttttggtgtccaacattctaagacctacg<br>aacatattgctggtttgttgttcgaaccatacgcttgagtgaatttactttaaatcttgcatttaaataaatttt<br>ctttttatagctttatgacttagtttcaatttatatactattttaatgacattttcgattcattgattgaaagctttg<br>tgtttttttcttgatgcgctattgcattgttcttgtctttttcgccacatgtaatatctgtagtagatacctgatac<br>attgtggatgctgagtgaaattttagttaataatggaggcgctcttaataattttggggatattggcttttttt<br>tttaaagtttacaaatgaattttttccgcca	
P_{TDH3}-<br>EfMvaE-<br>T_{TDH3}	gaataaaaaacacgctttttcagttcgagtttatcattatcaatactgccatttcaaagaatacgtaaataa<br>ttaatagtagtgattttcctaactttatttagtcaaaaaattagccttttaattctgctgtaacccgtacatgc<br>ccaaaataggggggcgggttacacagaatatataacatcgtaggtgtctgggtgaacagtttattcctg<br>gcatccactaaatataatggagcccgctttttaagctggcatccagaaaaaaaaagaatcccagcacc<br>aaaatattgttttcttcaccaaccatcagttcataggtccattctcttagcgcaactacagagaacaggg<br>gcacaaacaggcaaaaaacgggcacaacctcaatggagtgatgcaacctgcctggagtaaatgatg<br>acacaaggcaattgacccacgcatgtatctatctcattttcttacaccttctattaccttctgctctctctgat<br>ttggaaaaagctgaaaaaaaaggttgaaaccagttccctgaaattattcccctacttgactaataagtat<br>ataaagacggtaggtattgattgtaattctgtaaatctatttcttaaacttcttaaattctacttttatagttagt<br>ctttttttttagttttaaaacaccaagaacttagtttcgaataaacacacataaacaaacaaagtcgaatga<br>agaccgttgttatcattgatgctttgagaactccaatcggtaagtacaaaggttccttgtctcaagtttctg<br>ctgttgatttgggtactcatgttactactcagttgttgaagagacactccaccatctctgaagaaattgat<br>caagtcattttcggcaacgttttacaagctggtaatggtcaaaatccagctagacaaattgccatcaatt<br>ctggtttgtctcacgaaattccagctatgactgttaacgaagtttgtggttctggtatgaaggctgttatttt<br>ggctaaacagttgatccaattaggtgaagccgaagttttgattgctggtggtattgaaaacatgtctcaa<br>gctccaaagttgcagagattcaactacgaaactgaatcttacgatgccccattctcttctatgatgtatga<br>tggtttgaccgatgctttttctggtcaagctatgggtttgactgctgaaaatgttgctgaaaagtaccacg	*$P_{TDH3,}$<br>EfMvaE,<br>T_{TDH3}*

		ttaccagagaagaacaagatcaattctctgttcactctcagttgaaagctgctcaagctcaagctgaaggtatttttgctgacgaaattgctccattggaagtctctggtactttggttgaaaaagacgaaggtatcagaccaaactcctctgttgaaaaattgggcactttgaaaaccgtgttcaaagaagatggtactgttactgctggtaacgcttctactattaacgatggtgcttccgctttgattattgcctctcaagaatatgctgaagctcatggtttgccatacttggccattattagagattctgtcgaagttggtattgacccagcttatatgggtatttctccaatcaaggccatccaaaagttgttggctagaaatcagttgaccactgaagagattgacttgtacgaaatcaatgaagcctttgctgctacctctattgtcgttcaaagagaattggctttgccagaagaaaaggttaatatctatggtggtggtatctctttgggtcatgctattggtgctacaggtgctagattattgacttctttgtcctaccagctgaaccagaaagaaaaaaagtatggtgttgcctccttgtgtataggtggtggtttgggtttagctatgttgttggaaagaccacaacagaagaagaactccagattctatcaaatgtccccagaggaaagattggcctctttgttgaatgaaggtcaaatttccgctgacaccaagaaagagtttgaaaacactgctttgtcctctcaaattgctaaccacatgatcgagaaccagatttctgaaactgaagttccaatgggtgttggcttgcatttgacagttgacgaaacagattatttggtcccaatggctactgaagaaccatctgttattgctgcattgtctaacggtgctaaaattgctcaaggtttcaagaccgttaaccagcaaagattgatgagaggtcagatcgtttttacgatgttgcagatgccgaatccttgattgatgaattgcaagttagagaaaccgaaatcttccaacaagccgaattgtcttatccatctattgtcaaaagaggtggcggtttgagagacttgcagtatagagcttttgacgagtctttcgtttccgttgatttttggttgatgtcaaggatgctatgggtgccaatatcgttaatgctatgttggaaggtgttgccgagttgtttagagaatggtttgctgaacagaagatcctgttctccatttgtctaactacgctaccgaatctgttgttactatgaagactgctatcccagtttccagattgtccaaaggttctaacggtagagaaatcgctgaaaagatagttttggcttccagatatgcctctttggacccatacagagctgttactcataacaagggtattatgaacggtatcgaagcagttgttttggcaactggtaatgatacaagagcagtttcagcttcttgtcatgcttttgctgtaaaagaaggtagataccaaggtttgacatcttggactttggatggtgaacaactgattggtgaaatctctgttccattggctttagctacagttggtggtgctactaaggttttgccaaaatctcaagctgctgccgatttgttggctgttactgatgctaaagaattgtccagagttgttgctgctgttggtttagcacaaaacttggctgctttaagagctttggtttccgaaggtattcaaaaaggtcacatggcattgcaagctagatcattggctatgaccgttggtgccactggtaaagaagttgaagctgttgctcaacagttgaagaggcaaaagactatgaatcaagatagagccttggccatcttgaacgatttgagaaaacaatgaggccgcgtgaatttactttaaatcttgcatttaaataaattttctttttatagctttatgacttagtttcaatttatatactattttaatgacattttcgattcattgattgaaagctttgtgtttttcttgatgcgctattgcattgttcttgtctttttcgccacatgtaatatctgtagtagatacctgatacattgtggatgctgagtgaaattttagttaataatggaggcgctcttaataattttggggatattggcttttttttttaaagtttacaaatgaattttttccgccaggat	

P_{ADH1}-EfMvaS-T_{ADH1}	gggtgtacaatatggacttcctcttttctggcaaccaaacccatacatcgggattcctataataccttcgt<br>tggtctccctaacatgtaggtggcggaggggagatatacaatagaacagataccagacaagacataa<br>tgggctaaacaagactacaccaattacactgcctcattgatggtggtacataacgaactaatactgtag<br>ccctagacttgatagccatcatcatatcgaagtttcactaccctttttccatttgccatctattgaagtaata<br>ataggcgcatgcaacttcttttcttttttttcttttctctctcccccgttgttgtctcaccatatccgcaatga<br>caaaaaaatgatggaagacactaaaggaaaaaattaacgacaaagacagcaccaacagatgtcgtt<br>gttccagagctgatgaggggtatctcgaagcacacgaaactttttccttccttcattcacgcacactact<br>ctctaatgagcaacggtatacggccttccttccagttacttgaatttgaaataaaaaaaagtttgctgtctt<br>gctatcaagtataaatagacctgcaattattaatcttttgtttcctcgtcattgttctcgttccctttcttccttg<br>tttctttttctgcacaatatttcaagctataccaagcatacaatcaactatctcatatacacctagggtttatg<br>accattggcatcgacaagatttctttttcgttccaccttactacattgacatgactgctttggctgaagct<br>agaaatgttgatccaggtaagttccatattggtatcggtcaagatcaaatggctgttaacccaatttctca<br>agacatcgttacttttgctgctaatgctgctgaagccattttgacaaaagaggacaaagaagccattga<br>tatggttatcgttggcaccgaatcttccattgatgaatctaaagctgctgctgttgtcttgcatagattgat<br>gggtattcaaccattcgctaggtcctttgaaatcaaagaggcttgttacggcgctactgctggtttacaa<br>ttagctaaaaatcacgttgcattgcacccagataagaaggttttggttgttgctgctgatattgccaaata<br>cggtttaaattctggtggtgaacctactcaaggtgctggtgctgttgctatgttggttgcttctgaaccta<br>gaattttggccttgaaagaagataacgtcatgttgacacaagacatctacgatttttggaggccaactg<br>gtcatccatatccaatggttgatggtccattgtctaacgaaacctacattcaatctttcgcccaagtttgg<br>gatgaacacaaaaaaagaactggtttggatttcgctgattacgatgctttggcattccatattccatacac<br>taagatgggtaagaaggctttgttggctaagatttctgatcaaaccgaagccgaacaagaaagaatct<br>tggctagatacgaagagtccatcatctactcaagaagagtcggtaacttgtatactggctcattatactt<br>gggcctgatctctttgttggaaaacgctactactttgaccgccggtaatcaaattggtttgttttcttatggt<br>tcaggtgccgttgctgaatttttcactggtgaattggttgccggttatcaaaaccacttgcaaaaagaaa<br>ctcacttggccttgttggataacagaaccgaattgtctattgctgaatacgaagctatgttcgctgaaact<br>ttggataccgatattgaccaaaccttggaagatgagttgaagtattccatttccgccattaacaacaccg<br>ttaggtcttacagaaactaaaaacggccggccgcgaatttcttatgatttatgatttttattattaaataagt<br>tataaaaaaaataagtgtatacaaattttaaagtgactcttaggttttaaaacgaaaattcttattcttgagt<br>aactctttcctgtaggtcaggttgctttctcaggtatagcatgaggtcgctcttattgaccacacctctac<br>cggcatgccgagcaaatgcctgcaaatcgctccccatttcacccaattgtagatatgctaactccagc<br>aatgagttgatgaatctcggtgtgtattttatgtcctcagaggacaa	*P_{ADH1},<br>EfMvaS,<br>T_{ADH1}*
P_{TEF1}-PsBDH-T_{ADH1}	ccacacaccatagcttcaaaatgtttctactcctttttttactcttccagattttctcggactccgcgcatcgc<br>cgtaccacttcaaaacacccaagcacagcatactaaatttcccctctttcttcctctagggtgtcgttaatt<br>acccgtactaaaggtttggaaaagaaaaaagagaccgcctcgtttctttttcttcgtcgaaaaaggcaa<br>taaaaatttttatcacgtttctttttcttgaaaattttttttttgatttttttctctttcgatgacctcccattgatatt<br>taagttaataaacggtcttcaatttctcaagtttcagtttcatttttcttgttctattacaacttttttttacttcttg	*P_{TEF1},<br>PsBDH,<br>T_{ADH1}*

	ctcattagaaagaaagcatagcaatctaatctaagttttaattacaaaatgaagttgttggaaggtaaga<br>gaatcatcgttactggtggtgctcaaggtattggtgcttctgttgttagagcttatattgctgctggtgcta<br>cagttgcttctatggatatgaatgataccttgggtcaacaggttgtttctgaagctggtaaagctaatcca<br>ggttgtaaatccagatattaccattgcaacattgccgatagaccagaagttgaaaaggcttttgctactg<br>ctgctgaagatatgggtggtttggatgttatggttaatgttgctggtgttcatagacattctccaccagat<br>gctattgccgaagaattatacgatatgttgttcagggttaatgtcttgggtactattaacactaacgctgtt<br>gcttacagattgatgaaaggtcaaggtatcggtaacatcatcaacttcggttctgaatctggtttgactg<br>gtgaaatcaacaatgccttgtattctgctacaaaggctgctgttcatacttggactagaaatgttgctaga<br>caatggggtccagatggtattagaatcaatgctgttttgccttacatggtcaccccaatgtatgttgatttt<br>agaaacgccttgtcctccgaagatttggctgctcatgatgctgctactaagactgatattccattaggtg<br>gtaagtttggtgatgccgataaggatttggctccagttatggttttttggcttctgatgcttcccatttcat<br>gactggtcaaatgtttccagttgatggtggtttgattgccgttagatagcgcgccgaaataaattgaatt<br>gaattgaaatcgatagatcaattttttcttttctctttccccatcctttacgctaaaataatagtttattttatttt<br>ttgaatatttttatttatatacgtatatatagactattatttatcttttaatgattattaagatttttattaaaaaaa<br>aattcgctcctcttttaatgcctttatgcagtttttttttcccattcgatatttctatgttcgggttcagcgtattt<br>taagtttaataactcgaaaattctgcgttcgttaaagct	
P_{TEF1}-<br>$WvBAT4$ -<br>T_{ADH1}	ccacacaccatagcttcaaaatgtttctactcctttttactcttccagattttctcggactccgcgcatcgc<br>cgtaccacttcaaaacacccaagcacagcatactaaatttcccctctttcttcctctagggtgtcgttaatt<br>acccgtactaaaggtttggaaaagaaaaaagagaccgcctcgtttctttttcttcgtcgaaaaaggcaa<br>taaaaatttttatcacgtttctttttcttgaaaattttttttttgatttttttctctttcgatgacctcccattgatatt<br>taagttaataaacggtcttcaatttctcaagtttcagtttcatttttcttgttctattacaacttttttttacttcttg<br>ctcattagaaagaaagcatagcaatctaatctaagttttaattacaaaatgtctatggcttcttgggttaca<br>aaagttgctgctgaagaattggttgctccatgtgaacctactccatgtgctactttgccattgtcatctatt<br>gatcatgctttgggtttagccttcatggtcgaaatgatttctatctacccaaacaacaacagacaacatc<br>atcacccagctgctaaagttattcaagaagccttggctaaagccctggttccatattatccagttgctgg<br>tagattggtttcttctggtggtgattgtgttgaagttgcttgtaatggtgaaggtgtttggtttgttgaagct<br>atggttactgatcactctttgaacggtttgaacgattgggaatctatcccaagatcagttgttaaggatga<br>attgattccaggttgcccatctcatttgaagcaagaagaaatgatcatgatgatgcaagtcacccattttc<br>aatgtggtggttttatcgttggcttgaagttcaaccatttggttttcgatggtctaggtttcggtcaattcttg<br>aaagccattggtgaaattgcttgtggtagagcacatccatctgttgatccaatttggtacagagaagcta<br>ttccagttccattcatgctgtccaagtctatttctttccccattaccaaaattgacatcgtcaactcctcttac<br>gacttctctattcaaaccatcaagagattgaagggtcaaattgctaacgaaacctctaaccagttcacta<br>ccttcgaagttgttgttgctattttgtggaagtgtagaacccaagctattaacgctattggtgatgtttgttt<br>gtctttcccagccaacgttagatatttgttggatcaattacctaaggccggtggttattatggtaactgtat<br>ctacaacttgactgttaccgctacctctgaacaaatcaaaaaggcttcattggccgaattggtcagattg<br>attagaaacgccaaagaatccttgcctaccaagtttaaagaatggacttccggtaatttcaaagaggac	P_{TEF1},<br>$WvBAT4$,<br>T_{ADH1}

	ccttacaaaatctccagctcttacaactccttgatcttgtctgattggagatgcattgctattgatgaagct<br>gattttggttggggtttgccacattttgtttccccaattatgcacgatattccatttgcctctggtatcattttg<br>aagcagtctttgccaaaaggtggtgttcatttcgaaggtttggctactatgaaggaacatgagaagag<br>attcatcgacgagatcaacaagtttttggctaacgtcatgctgtgagcgaatttcttatgatttatgattttt<br>attattaaataagttataaaaaaaataagtgtatacaaattttaaagtgactcttaggttttaaaacgaaaat<br>tcttattcttgagtaactctttcctgtaggtcaggttgctttctcaggtatagcatgaggtcgctcttattga<br>ccacacctctaccggcatgccgagcaaatgcctgcaaatcgctccccatttcacccaattgtagatatg<br>ctaactccagcaatgagttgatgaatctcggtgtgtattttatgtcctcagaggacaa		

* Coding sequences are underlined.

Supplementary Table 2. Composition of SC medium in terms of amino acids and other nutrients.

**Composition**	**Amount (g)**
Adenine Sulfate (Adenine)	0.75
L-α-Alanine	2.00
L-Arginine	2.00
L-Asparagine monohydrate (L-Asparagine)	2.27
L-Aspartic acid	2.00
L-Cysteine	2.00
L-Glutamine	2.00
L-Glutamic acid	2.00
L-Glycine	2.00
myo-Inositol	2.00
L-Isoleucine	2.00
L-(+)-Lysine monohydrochloride	2.50
p-Aminobenzoic acid	2.00
L-Phenylalanine	2.00
L-Proline	2.00
L-Serine	2.00
L-Threonine	2.00
L-Tryptophan	2.00
L-Tyrosine	2.00
L-Valine	2.00
Total	39.52

Supplementary Note 1. Plasmid information.

**Name**	**pMT913**
**Sequence**	cctcaaagttactgacgaagcttatgatggtttggttattgttattggtagatggattcaagggtttttgtcccaaaagaataat<br>aacactattgaaggcttgaacttcatcagattacttgaaaaagattcaggtaaatctgagttcttattaagctacggtaagga<br>agtaaacaaaattccacaaagctacgaaaatttgaagaaaggttccactgtaaccagcaatgggttgaactgggaagtta<br>ttgaatatcacgcttaataaaggagaataaatcgttttctactttcttctgctgctataataagcacctatgggatctatatagta<br>tttttataacgatagactttataaaagaaaatacctaagtgaaaatttggtgaattttgagataattgttgggattccatttttaat<br>aaggcaataatattaggtatgtagaatatactagaagttctcctcgaggatttaggaatccataaaagggaatctgcaattc<br>tacacaattctataaatattattatcatcgttttatatgttaatattcattgatcctattacattatcaatccttgcgtttcagcttcca<br>ctaatttagatgactatttctcatcatttgcgtcatcttctaacaccgtatatgataatatactagtaacgtaaatactagttagta<br>gatgatagttgatttttattccaacactaagaaataatttcgccatttcttgaatgtatttaaagatatttaatgctataatagaca<br>tttaaatccaattcttccaacatacaatgggagtttggccgagtggtttaaggcgtcagatttaggtggatttaacctctaaa<br>atctctgatatcttcggatgcaagggttcgaatcccttagctctcattatttttgctttttctcttgaggtcacatgatcgcaaa<br>atggcaaatggcacgtgaagctgtcgatattggggaactgtggtggttggcaaatgactaattaagttagtcaaggcgcc<br>atcctcatgaaaactgtgtaacataataaccgaagtgtcgaaaaggtggcaccttgtccaattgaacacgctcgatgaaa<br>aaaataagatatatataaggttaagtaaagcgtctgttagaaaggaagtttttcctttttcttgctctcttgtcttttcatctactat<br>ttccttcgtgtaatacagggtcgtcagatacatagatacaattctattacccccatccatacaatgccatctcatttcgatact<br>gttcaactacacgccggccaagagaaccctggtgacaatgctcacagatccagagctgtaccaatttacgccaccactt<br>cttatgttttcgaaaactctaagcatggttcgcaattgtttggtctagaagttccaggttacgtctattcccgtttccaaaaccc<br>aaccagtaatgttttggaagaaagaattgctgctttagaaggtggtgctgctgctttggctgtttcctccggtcaagccgct<br>caaacccttgccatccaaggtttggcacacactggtgacaacatcgtttccacttcttacttatacggtggtacttataacca<br>gttcaaaatctcgttcaaaagatttggtatcgaggctagatttgttgaaggtgacaatccagaagaattcgaaaaggtcttt<br>gatgaaagaaccaaggctgtttatttggaaaccattggtaatccaaagtacaatgttccggattttgaaaaaattgttgcaat<br>tgctcacaaacacggtattccagttgtcgttgacaacacatttggtgccggtggttacttctgtcagccaattaaatacggtg<br>ctgatattgtaacacattctgctaccaaatggattggtggtcatggtactactatcggtggtattattgttgactctggtaagtt<br>cccatggaaggactacccagaaaagttccctcaattctctcaacctgccgaaggatatcacggtactatctacaatgaag<br>cctacggtaacttggcatacatcgttcatgttagaactgaactattaagagatttgggtccattgatgaacccatttgcctctt<br>tcttgctactacaaggtgttgaaacattatctttgagagctgaaagacacggtgaaaatgcattgaagttagccaaatggtt<br>agaacaatccccatacgtatcttgggtttcataccctggtttagcatctcattctcatcatgaaaatgctaagaagtatctatct<br>aacggtttcggtggtgtcttatctttcggtgtaaaagacttaccaaatgccgacaaggaaactgacccattcaaactttctg<br>gtgctcaagttgttgacaatttaaagcttgcctctaacttggccaatgttggtgatgccaagaccttagtcattgctccatact<br>tcactacccacaaacaattaaatgacaaagaaaagttggcatctggtgttaccaaggacttaattcgtgtctctgttggtatc<br>gaatttattgatgacattattgcagacttccagcaatcttttgaaactgttttcgctggccaaaaaccatgagtgtgcgtaatg<br>agttgtaaaattatgtataaacctactttctctcacaagtactatacttttataaaacgaactttattgaaatgaatatcctttttttc<br>ccttgttacatgtcgtgactcgtactttgaacctaaattgttctaacatcaaagaacagtgttaattcgcagtcgagaagtcg<br>agaaagatgccgatttgggcgcgaatcctttattttggcttcaccctcatactattatcagggccagaaaaaggaagtgttt<br>ccctccttcttgaattgatgttaccctcataaagcacgtggcctcttatcgagaaagaaattaccgtcgctcgtgatttgtttg<br>caaaaagaacaaaactgaaaaaacccagacacgctcgacttcctgtcttcctattgattgcagcttccaatttcgtcacac<br>aacaaggtcctagcgacggctcacaggttttgtaacaagcaatcgaaggttctggaatggcgggaaagggtttagtacc<br>acatgctatgatgcccactgtgatctccagagcaaagttcgttcgatcgtactgttactctctctctttcaaacagaattgtcc<br>gaatcgtgtgacaacaacagcctgttctcacacactcttttcttctaaccaagggggtggtttagtttagtagaacctcgtga<br>aacttacatttacatatatataaacttgcataaattggtcaatgcaagaaatacatatttggtcttttctaattcgtagtttttcaa<br>gttcttagatgctttctttttctctttttacagatcatcaaggaagtaattatctactttttacaacaaatataaaacgctagcgtc<br>gacatggacatcaagcaatctttgttgcatgctgccatgagattactgtctgctaaaggtagagatggtgctactatgagg<br>ccaatttgtgctgaagttggtgttactccaccaaccttgtgtcatcattatggtgacttgcaaggtttacacaaagctgctatt<br>gacgaaacctatagacaagttgctgaagcctatcatggtggtactgaagaaagaggtccattgaaaggtataagagatg<br>gttgggctactttcttgcaatttgcttactctgaacctaacatgtgcagaatgttggttcaacatattatggctggtgaaccac<br>catctatggttgctgatactttgagaggtgttgctgatgatttggctcaatttcatgctcaaggtagattgacttttccaccaag<br>agaagctgctcaattattgtggatgggtgctttaggtgctttgacttatgctttgtcaagagaaggtgcaggttacactcaag<br>atttggctttacaaaaggccaagttggatattaccttggttgccttgttcaacattgaagaagaaggtccagcagacgcttt<br>ggatgattttgatttggatatgttgcctgctgatgccttggatgacttcgacttagacatgttaccagccgatgcactagacg<br>attttgaccttggcatgttgccaggtccaccaaaaagaaaagaaaggtctaagatctgaaataaattgaattgaattgaaat<br>cgatagatcaattttttttcttttctctttccccatcctttacgctaaaataatagtttattttatttttgaatatttttatttatatacgta

	tatatagactattatttatcttttaatgattattaagatttttattaaaaaaaaaattcgctcctcttttaatgcctttatgcagttttttttt<br>cccattcgatatttctatgttcgggttcagcgtattttaagtttaataactcgaaaattctgcgttcgttaaagctgcggccgcc<br>accgcggtggagctccagcttttgttccctttagtgagggttaattgcgcgcttggcgtaatcatggtcatagctgtttcctg<br>tgtgaaattgttatccgctcacaattccacacaacatacgagccggaagcataaagtgtaaagcctggggtgcctaatga<br>gtgagctaactcacattaattgcgttgcgctcactgcccgctttccagtcgggaaacctgtcgtgccagctgcattaatga<br>atcggccaacgcgcggggagaggcggtttgcgtattgggcgctcttccgcttcctcgctcactgactcgctgcgctcgg<br>tcgttcggctgcggcgagcggtatcagctcactcaaaggcggtaatacggttatccacagaatcaggggataacgcag<br>gaaagaacatgtgagcaaaaggccagcaaaaggccaggaaccgtaaaaaggccgcgttgctggcgtttttccatagg<br>ctccgcccccctgacgagcatcacaaaaatcgacgctcaagtcagaggtggcgaaacccgacaggactataaagata<br>ccaggcgtttccccctggaagctccctcgtgcgctctcctgttccgaccctgccgcttaccggatacctgtccgcctttctc<br>ccttcgggaagcgtggcgctttctcatagctcacgctgtaggtatctcagttcggtgtaggtcgttcgctccaagctgggc<br>tgtgtgcacgaaccccccgttcagcccgaccgctgcgccttatccggtaactatcgtcttgagtccaacccggtaagaca<br>cgacttatcgccactggcagcagccactggtaacaggattagcagagcgaggtatgtaggcggtgctacagagttcttg<br>aagtggtggcctaactacggctacactagaagaacagtatttggtatctgcgctctgctgaagccagttaccttcggaaaa<br>agagttggtagctcttgatccggcaaacaaaccaccgctggtagcggtggtttttttgtttgcaagcagcagattacgcgc<br>agaaaaaaaggatctcaagaagatcctttgatcttttctacggggtctgacgctcagtggaacgaaaactcacgttaagg<br>gattttggtcatgagattatcaaaaaggatcttcacctagatccttttaaattaaaaatgaagttttaaatcaatctaaagtatat<br>atgagtaaacttggtctgacagttaccaatgcttaatcagtgaggcacctatctcagcgatctgtctatttcgttcatccatag<br>ttgcctgactccccgtcgtgtagataactacgatacgggagggcttaccatctggccccagtgctgcaatgataccgcga<br>gacccacgctcaccggctccagatttatcagcaataaaccagccagccggaagggccgagcgcagaagtggtcctgc<br>aactttatccgcctccatccagtctattaattgttgccgggaagctagagtaagtagttcgccagttaatagtttgcgcaacg<br>ttgttgccattgctacaggcatcgtggtgtcacgctcgtcgtttggtatggcttcattcagctccggttcccaacgatcaagg<br>cgagttacatgatcccccatgttgtgcaaaaaagcggttagctccttcggtcctccgatcgttgtcagaagtaagttggcc<br>gcagtgttatcactcatggttatggcagcactgcataattctcttactgtcatgccatccgtaagatgcttttctgtgactggt<br>gagtactcaaccaagtcattctgagaatagtgtatgcggcgaccgagttgctcttgcccggcgtcaatacgggataatac<br>cgcgccacatagcagaactttaaaagtgctcatcattggaaaacgttcttcggggcgaaaactctcaaggatcttaccgct<br>gttgagatccagttcgatgtaacccactcgtgcacccaactgatcttcagcatcttttactttcaccagcgtttctgggtgag<br>caaaaacaggaaggcaaaatgccgcaaaaaagggaataagggcgacacggaaatgttgaatactcatactcttcctttt<br>tcaatattattgaagcatttatcagggttattgtctcatgagcggatacatatttgaatgtatttagaaaaataaacaaatagg<br>ggttccgcgcacatttccccgaaaagtgccacctg

**Name**	**pKZ031**
**Sequence**	tcgcgcgtttcggtgatgacggtgaaaacctctgacacatgcagctcccggagacggtcacagcttgtctgtaagcgga<br>tgccgggagcagacaagcccgtcagggcgcgtcagcgggtgttggcgggtgtcggggctggcttaactatgcggcat<br>cagagcagattgtactgagagtgcaccataaattcctattgtgagggtcagttatttcatccagatataacccgagaggaa<br>acttcttagcgtctgttttcgtaccataaggcagttcatgaggtatattttcgttattgaagcccagctcgtgaatgcttaatgc<br>tgctgaactggtgtccatgtcgcctagctacgcaatctccacaggctgcaaaggttttgtctcaagagcaatgttattgtgc<br>accccgtaattggtcaacaagtttaatctgtgcttgtccaccagctctgtcgtaaccttcagttcatcgactatctgaagaaat<br>ttactaggaatagtgccatggtacagcaaccgagaatggcaatttctactcgggttcagcaacgctgcataaacgctgttg<br>gtgccgtagacatattcgaagataggattatcattcataagtttcagagcaatgtccttattctggaacttggatttatggctct<br>tttggtttaatttcgcctgattcttgatctcctttagcttctcgacgtgggcctttttcttgccatatggatccgctgcacggtcct<br>gttccctagcatgtacgtgagcgtatttccttttaaaccacgacgctttgtcttcattcaacgtttcccattgtttttttctactatt<br>gctttgctgtgggaaaaacttatcgaaagatgacgactttttcttaattctcgttttaagagcttggtgagcgctaggagtca<br>ctgccaggtatcgtttgaacacggcattagtcagggaagtcataacacagtcctttcccgcaattttctttttctattactcttg<br>gcctcctctagtacactctatattttttatgcctcggtaatgattttcattttttttttccacctagcggatgactcttttttttctta<br>gcgattggcattatcacataatgaattatacattatataaagtaatgtgatttcttcgaagaatatactaaaaaatgagcaggc<br>aagataaacgaaggcaaagatgacagagcagaaagccctagtaaagcgtattacaaatgaaaccaagattcagattgc<br>gatctctttaaagggtggtcccctagcgatagagcactcgatcttcccagaaaaagaggcagaagcagtagcagaaca<br>ggccacacaatcgcaagtgattaacgtccacacaggtatagggtttctggaccatatgatacatgctctggccaagcatt<br>ccggctggtcgctaatcgttgagtgcattggtgacttacacatagacgaccatcacaccactgaagactgcgggattgct<br>ctcggtcaagcttttaaagaggcgctaggggccgtgcgtggagtaaaaaggtttggatcaggatttgcgcctttggatga<br>ggcactttccagagcggtggtagatctttcgaacaggccgtacgcagttgtcgaacttggtttgcaaagggagaaagta<br>ggagatctctcttgcgagatgatcccgcattttcttgaaagctttgcagaggctagcagaattaccctccacgttgattgtct<br>gcgaggcaagaatgatcatcaccgtagtgagagtgcgttcaaggctcttgcggttgccataagagaagccacctcgcc<br>caatggtaccaacgatgttccctccaccaaaggtgttcttatgtagtgacaccgattatttaaagctgcagcatacgatatat<br>atacatgtgtatatatgtatacctatgaatgtcagtaagtatgtatacgaacagtatgatactgaagatgacaaggtaatgca<br>tcattctatacgtgtcattctgaacgaggcgcgctttccttttttctttttgctttttctttttttttctcttgaactcgacggatctatg<br>cggtgtgaaataccgcacagatgcgtaaggagaaaataccgcatcaggaaattgtaagcgttaatattttgttaaaattcg<br>cgttaaatttttgttaaatcagctcattttttaaccaataggccgaaatcggcaaaatcccttataaatcaaaagaatagaccg<br>agatagggttgagtgttgttccagtttggaacaagagtccactattaaagaacgtggactccaacgtcaaagggcgaaaa<br>accgtctatcagggcgatggcccactacgtgaaccatcaccctaatcaagttttttggggtcgaggtgccgtaaagcact<br>aaatcggaaccctaaagggagcccccgatttagagcttgacggggaaagccggcgaacgtggcgagaaaggaagg<br>gaagaaagcgaaaggagcgggcgctagggcgctggcaagtgtagcggtcacgctgcgcgtaaccaccacacccgc<br>cgcgcttaatgcgccgctacagggcgcgtccattcgccattcaggctgcgcaactgttgggaagggcgatcggtgcgg<br>gcctcttcgctattacgccagctggcgaaagggggatgtgctgcaaggcgattaagttgggtaacgccagggttttccc<br>agtcacgacgttgtaaaacgacggccagtgagcgcgcgtaatacgactcactatagggcgaattgggtaccgggccc<br>cccctcgagttgtcctctgaggacataaaatacacaccgagattcatcaactcattgctggagttagcatatctacaattgg<br>gtgaaatggggagcgatttgcaggcatttgctcggcatgccggtagaggtgtggtcaataagagcgacctcatgctata<br>cctgagaaagcaacctgacctacaggaaagagttactcaagaataagaattttcgttttaaaacctaagagtcactttaaa<br>atttgtatacacttattttttttataacttatttaataataaaaatcataaatcataagaaattcgcggccggccgtttttagtttctg<br>taagacctaacggtgttgttaatggcggaaatggaatacttcaactcatcttccaaggtttggtcaatatcggtatccaaagt<br>ttcagcgaacatagcttcgtattcagcaatagacaattcggttctgttatccaacaaggccaagtgagtttctttttgcaagtg<br>gttttgataaccggcaaccaattcaccagtgaaaaattcagcaacggcacctgaaccataagaaaacaaaccaatttgat<br>taccggcggtcaaagtagtagcgttttccaacaaagagatcaggcccaagtataatgagccagtatacaagttaccgact<br>cttcttgagtagatgatggactcttcgtatctagccaagattctttcttgttcggcttcggtttgatcagaaatcttagccaaca<br>aagccttcttacccatcttagtgtatggaatatggaatgccaaagcatcgtaatcagcgaaatccaaaccagttcttttttgt<br>gttcatcccaaacttgggcgaaagattgaatgtaggtttcgttagacaatggaccatcaaccattggatatggatgaccag<br>ttggcctccaaaaatcgtagatgtcttgtgtcaacatgacgttatcttctttcaaggccaaaattctaggttcagaagcaacc<br>aacatagcaacagcaccagcaccttgagtaggttcaccaccagaatttaaaccgtatttggcaatatcagcagcaacaac<br>caaaaccttcttatctgggtgcaatgcaacgtgatttttagctaattgtaaaccagcagtagcgccgtaacaagcctctttga<br>tttcaaaggacctagcgaatggttgaatacccatcaatctatgcaagacaacagcagcagctttagattcatcaatggaag<br>attcggtgccaacgataaccatatcaatggcttctttgtcctcttttgtcaaaatggcttcagcagcattagcagcaaaagta<br>acgatgtcttgagaaattgggttaacagccatttgatcttgaccgataccaatatggaacttacctggatcaacatttctagc<br>ttcagccaaagcagtcatgtcaatgtagtaaggtggaacgaaaaaagaaatcttgtcgatgccaatggtcataaacccta<br>ggtgtatatgagatagttgattgtatgcttggtatagcttgaaatattgtgcagaaaaagaaacaaggaagaaagggaac

	gagaacaatgacgaggaaacaaaagattaataattgcaggtctatttatacttgatagcaagacagcaaacttttttttatttc<br>aaattcaagtaactggaaggaaggccgtataccgttgctcattagagagtagtgtgcgtgaatgaaggaaggaaaaagt<br>ttcgtgtgcttcgagataccccctcatcagctctggaacaacgacatctgttggtgctgtctttgtcgttaattttttcctttagtg<br>tcttccatcatttttttgtcattgcggatatggtgagacaacaacgggggagagagaaaagaaaaaaaaagaaaagaagt<br>tgcatgcgcctattattacttcaatagatggcaaatggaaaaagggtagtgaaacttcgatatgatgatggctatcaagtct<br>agggctacagtattagttcgttatgtaccaccatcaatgaggcagtgtaattggtgtagtcttgtttagcccattatgtcttgtc<br>tggtatctgttctattgtatatctcccctccgccacctacatgttagggagaccaacgaaggtattataggaatcccgatgta<br>tgggtttggttgccagaaaagaggaagtccatattgtacacccccgcgggaataaaaaacacgctttttcagttcgagttt<br>atcattatcaatactgccatttcaaagaatacgtaaataattaatagtagtgattttcctaactttatttagtcaaaaaattagcct<br>tttaattctgctgtaacccgtacatgcccaaaataggggggcgggttacacagaatatataacatcgtaggtgtctgggtga<br>acagtttattcctggcatccactaaatataatggagcccgctttttaagctggcatccagaaaaaaaaagaatcccagcac<br>caaaatattgttttcttcaccaaccatcagttcataggtccattctcttagcgcaactacagagaacaggggcacaaacag<br>gcaaaaaacgggcacaacctcaatggagtgatgcaacctgcctggagtaaatgatgacacaaggcaattgacccacg<br>catgtatctatctcattttcttacaccttctattaccttctgctctctctgatttggaaaaagctgaaaaaaaaggttgaaacca<br>gttccctgaaattattcccctacttgactaataagtatataaagacggtaggtattgattgtaattctgtaaatctatttcttaaa<br>cttcttaaattctacttttatagttagtcttttttttagttttaaaacaccaagaacttagtttcgaataaacacacataaacaaaca<br>aagtcgaatgaagaccgttgttatcattgatgctttgagaactccaatcggtaagtacaaaggttccttgtctcaagtttctg<br>ctgttgatttgggtactcatgttactactcagttgttgaagagacactccaccatctctgaagaaattgatcaagtcattttcg<br>gcaacgttttacaagctggtaatggtcaaaatccagctagacaaattgccatcaattctggtttgtctcacgaaattccagct<br>atgactgttaacgaagtttgtggttctggtatgaaggctgttattttggctaaacagttgatccaattaggtgaagccgaagtt<br>ttgattgctggtggtattgaaaacatgtctcaagctccaaagttgcagagattcaactacgaaactgaatcttacgatgccc<br>cattctcttctatgatgtatgatggtttgaccgatgctttttctggtcaagctatgggtttgactgctgaaaatgttgctgaaaa<br>gtaccacgttaccagagaagaacaagatcaattctctgttcactctcagttgaaagctgctcaagctcaagctgaaggtat<br>tttgctgacgaaattgctccattggaagtctctggtactttggttgaaaaagacgaaggtatcagaccaaactcctctgttg<br>aaaaattgggcactttgaaaaccgtgttcaaagaagatggtactgttactgctggtaacgcttctactattaacgatggtgct<br>tccgctttgattattgcctctcaagaatatgctgaagctcatggtttgccatacttggccattattagagattctgtcgaagttg<br>gtattgacccagcttatatgggtatttctccaatcaaggccatccaaaagttgttggctagaaatcagttgaccactgaaga<br>gattgacttgtacgaaatcaatgaagcctttgctgctacctctattgtcgttcaaagagaattggctttgccagaagaaaag<br>gttaatatctatggtggtggtatctctttgggtcatgctattggtgctacaggtgctagattattgacttctttgtcctaccagct<br>gaaccagaaagaaaaaaagtatggtgttgcctccttgtgtataggtggtggtttgggtttagctatgttgttggaaagacca<br>caacagaagaagaactccagattctatcaaatgtccccagaggaaagattggcctctttgttgaatgaaggtcaaatttcc<br>gctgacaccaagaaagagtttgaaaacactgctttgtcctctcaaattgctaaccacatgatcgagaaccagatttctgaa<br>actgaagttccaatgggtgttggcttgcatttgacagttgacgaaacagattatttggtcccaatggctactgaagaaccat<br>ctgttattgctgcattgtctaacggtgctaaaattgctcaaggtttcaagaccgttaaccagcaaagattgatgagaggtca<br>gatcgtttttttacgatgttgcagatgccgaatccttgattgatgaattgcaagttagagaaaccgaaatcttccaacaagcc<br>gaattgtcttatccatctattgtcaaaagaggtggcggtttgagagacttgcagtatagagcttttgacgagtctttcgtttcc<br>gttgatttttttggttgatgtcaaggatgctatgggtgccaatatcgttaatgctatgttggaaggtgttgccgagttgtttagag<br>aatggtttgctgaacagaagatcctgttctccattttgtctaactacgctaccgaatctgttgttactatgaagactgctatccc<br>agtttccagattgtccaaaggttctaacggtagagaaatcgctgaaaagatagttttggcttccagatatgcctctttggacc<br>catacagagctgttactcataacaagggtattatgaacggtatcgaagcagttgttttggcaactggtaatgatacaagag<br>cagtttcagcttcttgtcatgcttttgctgtaaaagaaggtagataccaaggtttgacatcttggactttggatggtgaacaac<br>tgattggtgaaatctctgttccattggctttagctacagttggtggtgctactaaggttttgccaaaatctcaagctgctgccg<br>atttgttggctgttactgatgctaaagaattgtccagagttgttgctgctgttggtttagcacaaaacttggctgctttaagag<br>ctttggtttccgaaggtattcaaaaaggtcacatggcattgcaagctagatcattggctatgaccgttggtgccactggtaa<br>agaagttgaagctgttgctcaacagttgaagaggcaaaagactatgaatcaagatagagccttggccatcttgaacgattt<br>gagaaaacaatgaggccgcgtgaatttactttaaatcttgcatttaaataaattttctttttatagctttatgacttagtttcaattt<br>atatactattttaatgacattttcgattcattgattgaaagctttgtgtttttttcttgatgcgctattgcattgttcttgtctttttcgcc<br>acatgtaatatctgtagtagatacctgatacattgtggatgctgagtgaaattttagttaataatggaggcgctcttaataattt<br>tggggatattggcttttttttttaaagtttacaaatgaatttttttccgccaggatttaattaaaaagatgccgatttgggcgcgaa<br>tcctttattttggcttcaccctcatactattatcagggccagaaaaaggaagtgtttccctccttcttgaattgatgttaccctca<br>taaagcacgtggcctcttatcgagaaagaaattaccgtcgctcgtgatttgtttgcaaaaagaacaaaactgaaaaaacc<br>cagacacgctcgacttcctgtcttcctattgattgcagcttccaatttcgtcacacaacaaggtcctagcgacggctcacag<br>gttttgtaacaagcaatcgaaggttctggaatggcgggaaagggtttagtaccacatgctatgatgcccactgtgatctcc<br>agagcaaagttcgttcgatcgtactgttactctctctctttcaaacagaattgtccgaatcgtgtgacaacaacagcctgttc

	tcacacactcttttcttctaaccaagggggtggtttagtttagtagaacctcgtgaaacttacatttacatatatataaacttgc<br>ataaattggtcaatgcaagaaatacatatttggtcttttctaattcgtagtttttcaagttcttagatgctttctttttctcttttttaca<br>gatcatcaaggaagtaattatctactttttacaacaaatataaaaccccgggggcgcgccgaaataaattgaattgaattga<br>aatcgatagatcaatttttttcttttctctttccccatcctttacgctaaaataatagtttattttattttttgaatatttttttatttatatac<br>gtatatatagactattatttatcttttaatgattattaagatttttattaaaaaaaaattcgctcctcttttaatgcctttatgcagtttt<br>tttttcccattcgatatttctatgttcgggttcagcgtattttaagtttaataactcgaaaattctgcgttcgttaaagctgagctc<br>cagcttttgttccctttagtgagggttaattgcgcgcttggcgtaatcatggtcatagctgtttcctgtgtgaaattgttatccg<br>ctcacaattccacacaacatacgagccggaagcataaagtgtaaagcctggggtgcctaatgagtgagctaactcacat<br>taattgcgttgcgctcactgcccgctttccagtcgggaaacctgtcgtgccagctgcattaatgaatcggccaacgcgcg<br>gggagaggcggtttgcgtattgggcgctcttccgcttcctcgctcactgactcgctgcgctcggtcgttcggctgcggcg<br>agcggtatcagctcactcaaaggcggtaatacggttatccacagaatcaggggataacgcaggaaagaacatgtgagc<br>aaaaggccagcaaaaggccaggaaccgtaaaaaggccgcgttgctggcgtttttccataggctccgcccccctgacg<br>agcatcacaaaaatcgacgctcaagtcagaggtggcgaaacccgacaggactataaagataccaggcgtttccccctg<br>gaagctccctcgtgcgctctcctgttccgaccctgccgcttaccggatacctgtccgcctttctcccttcgggaagcgtgg<br>cgctttctcatagctcacgctgtaggtatctcagttcggtgtaggtcgttcgctccaagctgggctgtgtgcacgaacccc<br>ccgttcagcccgaccgctgcgccttatccggtaactatcgtcttgagtccaacccggtaagacacgacttatcgccactg<br>gcagcagccactggtaacaggattagcagagcgaggtatgtaggcggtgctacagagttcttgaagtggtggcctaact<br>acggctacactagaagaacagtatttggtatctgcgctctgctgaagccagttaccttcggaaaaagagttggtagctctt<br>gatccggcaaacaaaccaccgctggtagcggtggtttttttgtttgcaagcagcagattacgcgcagaaaaaaaggatct<br>caagaagatcctttgatcttttctacggggtctgacgctcagtggaacgaaaactcacgttaagggattttggtcatgagat<br>tatcaaaaaggatcttcacctagatccttttaaattaaaaatgaagttttaaatcaatctaaagtatatatgagtaaacttggtct<br>gacagttaccaatgcttaatcagtgaggcacctatctcagcgatctgtctatttcgttcatccatagttgcctgactccccgt<br>cgtgtagataactacgatacgggagggcttaccatctggccccagtgctgcaatgataccgcgagacccacgctcacc<br>ggctccagatttatcagcaataaaccagccagccggaagggccgagcgcagaagtggtcctgcaactttatccgcctc<br>catccagtctattaattgttgccgggaagctagagtaagtagttcgccagttaatagtttgcgcaacgttgttgccattgcta<br>caggcatcgtggtgtcacgctcgtcgtttggtatggcttcattcagctccggttcccaacgatcaaggcgagttacatgat<br>cccccatgttgtgcaaaaaagcggttagctccttcggtcctccgatcgttgtcagaagtaagttggccgcagtgttatcact<br>catggttatggcagcactgcataattctcttactgtcatgccatccgtaagatgcttttctgtgactggtgagtactcaaccaa<br>gtcattctgagaatagtgtatgcggcgaccgagttgctcttgcccggcgtcaatacgggataataccgcgccacatagca<br>gaactttaaaagtgctcatcattggaaaacgttcttcggggcgaaaactctcaaggatcttaccgctgttgagatccagttc<br>gatgtaacccactcgtgcacccaactgatcttcagcatcttttactttcaccagcgtttctgggtgagcaaaaacaggaag<br>gcaaaatgccgcaaaaaagggaataagggcgacacggaaatgttgaatactcatactcttcctttttcaatattattgaag<br>catttatcagggttattgtctcatgagcggatacatatttgaatgtatttagaaaaataaacaaataggggttccgcgcacatt<br>tccccgaaaagtgccacctgacgtctaagaaaccattattatcatgacattaacctataaaaataggcgtatcacgaggcc<br>ctttcgtc

Name	pKZ034
Sequence	tcgcgcgtttcggtgatgacggtgaaaacctctgacacatgcagctcccggagacggtcacagcttgtctgtaagcgga<br>tgccgggagcagacaagcccgtcagggcgcgtcagcgggtgttggcgggtgtcggggctggcttaactatgcggcat<br>cagagcagattgtactgagagtgcaccataccaccttttcaattcatcatttttttttttattcttttttttgatttcggtttccttgaa<br>attttttgattcggtaatctccgaacagaaggaagaacgaaggaaggagcacagacttagattggtatatatacgcatat<br>gtagtgttgaagaaacatgaaattgcccagtattcttaacccaactgcacagaacaaaaacctgcaggaaacgaagata<br>aatcatgtcgaaagctacatataaggaacgtgctgctactcatcctagtcctgttgctgccaagctatttaatatcatgcacg<br>aaaagcaaacaaacttgtgtgcttcattggatgttcgtaccaccaaggaattactggagttagttgaagcattaggtcccaa<br>aatttgtttactaaaaacacatgtggatatcttgactgatttttccatggagggcacagttaagccgctaaaggcattatccg<br>ccaagtacaattttttactcttcgaagacagaaaatttgctgacattggtaatacagtcaaattgcagtactctgcgggtgtat<br>acagaatagcagaatgggcagacattacgaatgcacacggtgtggtgggcccaggtattgttagcggtttgaagcagg<br>cggcagaagaagtaacaaaggaacctagaggccttttgatgttagcagaattgtcatgcaagggctccctatctactgga<br>gaatatactaagggtactgttgacattgcgaagagcgacaaagattttgttatcggctttattgctcaaagagacatgggtg<br>gaagagatgaaggttacgattggttgattatgacacccggtgtgggtttagatgacaagggagacgcattgggtcaaca<br>gtatagaaccgtggatgatgtggtctctacaggatctgacattattattgttggaagaggactatttgcaaagggaaggga<br>tgctaaggtagagggtgaacgttacagaaaagcaggctgggaagcatatttgagaagatgcggccagcaaaactaaa<br>aaactgtattataagtaaatgcatgtatactaaactcacaaattagagcttcaatttaattatatcagttattacccgcgaatct<br>cggtcgtaatgatttctataatgacgaaaaaaaaaaaattggaaagaaaaagcttcatggcctttataaaaaggaactatc<br>caatacctcgccagaaccaagtaacagtattttacggggcacaaatcaagaacaataagacaggactgtaaagtagga<br>cgcattgaactccaaagaacaacaagagttccaaaaagtagtggaacaaaagcaaatgaaggatttcatgcgtttgtact<br>ctaatctggtagaaagatgtttcacagactgtgtcaatgacttcacaacatcaaagctaaccaataaggaacaaacatgca<br>tcatgaagtgctcagaaaagttcttgaagcatagcgaacgtgtagggcagcgtttccaagaacaaaacgctgccttggg<br>acaaggcttgggccgataaggtgtactggcgtatatatatctaattatgtatctctggtgtagcccatttttagcatgtaaata<br>taaagagaaaccatatctaatctaaccaaatccaaacaaaattcaatagttactatcgctttttttctttctgtatcgcaaataag<br>tgaaaattaaaaaagaaagattaaattggaagttggatatgggctggaacagcagcagtaatcggtatcgggttcgccac<br>taatgacgtcctacgattgcactcaacagaccttgacgctcacgccgtagcgggcgacaagtcaaacggaacaaccgtt<br>gccgttcccatcggagtccgatatgcggtgtgaaataccgcacagatgcgtaaggagaaaataccgcatcaggaaatt<br>gtaagcgttaatattttgttaaaattcgcgttaaatttttgttaaatcagctcattttttaaccaataggccgaaatcggcaaaat<br>cccttataaatcaaaagaatagaccgagatagggttgagtgttgttccagtttggaacaagagtccactattaaagaacgt<br>ggactccaacgtcaaagggcgaaaaaccgtctatcagggcgatggcccactacgtgaaccatcaccctaatcaagtttt<br>ttggggtcgaggtgccgtaaagcactaaatcggaaccctaaagggagcccccgatttagagcttgacggggaaagcc<br>ggcgaacgtggcgagaaaggaagggaagaaagcgaaaggagcgggcgctagggcgctggcaagtgtagcggtca<br>cgctgcgcgtaaccaccacacccgccgcgcttaatgcgccgctacagggcgcgtccattcgccattcaggctgcgcaa<br>ctgttgggaagggcgatcggtgcgggcctcttcgctattacgccagcggatcccaggctctatatctgcgatatactgag<br>catgacaggctctatatctgcgatatactgagcatcacaggctctatatctgcgatatactgagcatcacaggctctatatct<br>gcgatatactgagcatgccaggctctatatctgcgatatactgagcatagcaggctctatatctgcgatatactgagcatca<br>caggctctatatctgcgatatactgagcatcacaggctctatatctgcgatatactgagcatgccaggctctatatctgcga<br>tatactgagcatagcaggctctatatctgcgatatactgagaatacgtatgtgatccggatgataatgcgattagtttttttagc<br>cttatttctggggtaattaatcagcgaagcgatgatttttgatctattaacagatatataaatgcaaaaactgcataaccacttt<br>aactaatactttcaacattttcggtttgtattacttcttattcaaatgtaataaaagtatcaacaaaaaattgttaatatacctctat<br>actttaacgtcaaggagaaaaaactatactgcagatggtcagtgtcatcaaagaagaaatgaagatcaagttgcacatgg<br>aaggtaacgttaatggtcatgcctttgttattgaaggtgatggtaaaggtaaaccatacgatggtactcaaactttgaacttg<br>actgtcaaagaaggtgctccattgccattctcttacgatattttgactaacgccttccaatacggtaatagagcttttactaag<br>tacccagccgatatcccagattactttaagcaaacttttccagaaggttactcctgggaaagaactatgtcttacgaagata<br>acgctatctgcaacgtcagatccgaaatttctatggaaggtgattgcttcatctacaagatcagattcgatggtaagaacttt<br>ccaccaaatggtccagtcatgcaaaaaaagactttgaagtgggaaccatccaccgaaatgatgtatgttagagatggttt<br>cttgatgggtgatgtcaatatggctttgttgttggaaggtggtggtcatcatagatgtgatttcaagacttcttacaaggcca<br>agaaggttgttcaattgccagatgctcataagatcgatcacagaatcgaaatcttgtcccacgatagagattactccaagg<br>ttaagttgtacgaaaacgctgttgctagaaactctttgttgccatctcaagcttctaagtaagtcgacctcgagtcatgtaatt<br>agttatgtcacgcttacattcacgccctccccccacatccgctctaaccgaaaaggaaggagttagacaacctgaagtct<br>aggtccctatttatttttttatagttatgttagtattaagaacgttatttatatttcaaatttttcttttttttctgtacagacgcgtgtac<br>gcatgtaacattatactgaaaaccttgcttgagaaggttttgggacgctcgaaggctttaatttgcggccgccaccgcggt

	ggagctccagcttttgttccctttagtgagggttaattgcgcgcttggcgtaatcatggtcatagctgtttcctgtgtgaaatt<br>gttatccgctcacaattccacacaacatacgagccggaagcataaagtgtaaagcctggggtgcctaatgagtgagcta<br>actcacattaattgcgttgcgctcactgcccgctttccagtcgggaaacctgtcgtgccagctgcattaatgaatcggcca<br>acgcgcggggagaggcggtttgcgtattgggcgctcttccgcttcctcgctcactgactcgctgcgctcggtcgttcggc<br>tgcggcgagcggtatcagctcactcaaaggcggtaatacggttatccacagaatcaggggataacgcaggaaagaac<br>atgtgagcaaaaggccagcaaaaggccaggaaccgtaaaaaggccgcgttgctggcgtttttccataggctccgcccc<br>cctgacgagcatcacaaaaatcgacgctcaagtcagaggtggcgaaacccgacaggactataaagataccaggcgttt<br>ccccctggaagctccctcgtgcgctctcctgttccgaccctgccgcttaccggatacctgtccgcctttctcccttcggga<br>agcgtggcgctttctcatagctcacgctgtaggtatctcagttcggtgtaggtcgttcgctccaagctgggctgtgtgcac<br>gaaccccccgttcagcccgaccgctgcgccttatccggtaactatcgtcttgagtccaacccggtaagacacgacttatc<br>gccactggcagcagccactggtaacaggattagcagagcgaggtatgtaggcggtgctacagagttcttgaagtggtg<br>gcctaactacggctacactagaagaacagtatttggtatctgcgctctgctgaagccagttaccttcggaaaaagagttgg<br>tagctcttgatccggcaaacaaaccaccgctggtagcggtggtttttttgtttgcaagcagcagattacgcgcagaaaaaa<br>aggatctcaagaagatcctttgatcttttctacggggtctgacgctcagtggaacgaaaactcacgttaagggattttggtc<br>atgagattatcaaaaaggatcttcacctagatccttttaaattaaaaatgaagttttaaatcaatctaaagtatatatgagtaaa<br>cttggtctgacagttaccaatgcttaatcagtgaggcacctatctcagcgatctgtctatttcgttcatccatagttgcctgac<br>tccccgtcgtgtagataactacgatacgggagggcttaccatctggccccagtgctgcaatgataccgcgagacccacg<br>ctcaccggctccagatttatcagcaataaaccagccagccggaagggccgagcgcagaagtggtcctgcaactttatc<br>cgcctccatccagtctattaattgttgccgggaagctagagtaagtagttcgccagttaatagtttgcgcaacgttgttgcc<br>attgctacaggcatcgtggtgtcacgctcgtcgtttggtatggcttcattcagctccggttcccaacgatcaaggcgagtta<br>catgatcccccatgttgtgcaaaaaagcggttagctccttcggtcctccgatcgttgtcagaagtaagttggccgcagtgtt<br>atcactcatggttatggcagcactgcataattctcttactgtcatgccatccgtaagatgcttttctgtgactggtgagtactc<br>aaccaagtcattctgagaatagtgtatgcggcgaccgagttgctcttgcccggcgtcaatacgggataataccgcgcca<br>catagcagaactttaaaagtgctcatcattggaaaacgttcttcggggcgaaaactctcaaggatcttaccgctgttgagat<br>ccagttcgatgtaacccactcgtgcacccaactgatcttcagcatcttttactttcaccagcgtttctgggtgagcaaaaac<br>aggaaggcaaaatgccgcaaaaaagggaataagggcgacacggaaatgttgaatactcatactcttcctttttcaatatta<br>ttgaagcatttatcagggttattgtctcatgagcggatacatatttgaatgtatttagaaaaataaacaaataggggttccgc<br>gcacatttccccgaaaagtgccacctgacgtctaagaaaccattattatcatgacattaacctataaaaataggcgtatcac<br>gaggccctttcgtc

Name	pKZ045
Sequence	tcgcgcgtttcggtgatgacggtgaaaacctctgacacatgcagctcccggagacggtcacagcttgtctgtaagcgga<br>tgccgggagcagacaagcccgtcagggcgcgtcagcgggtgttggcgggtgtcggggctggcttaactatgcggcat<br>cagagcagattgtactgagagtgcaccatagtcaatgaaatgaacattgatttactatcaatttcttctcacaagatttacgg<br>tccaaagggaataggtgccatctatgtaagaaggagaccaagagttagattagaacctttactatccggtggtggccaag<br>agagaggtttaagatctggtactttggcccccccattggtagcgggatttggtgaagctgcgagattgatgaagaaagaa<br>tttgacaacgatcaagctcacatcaaaagactatccgataaattagtcaaaggtctattatccgctgaacataccacgttga<br>acggatctccagatcatcgttatccagggtgtgttaacgtttctttcgcctacgtggaaggagaatctttattgatggcacta<br>agggatatcgcattatcctcgggttcagcctgtacatctgcttccctagaaccttcttatgttttacatgcgctgggtaaggat<br>gatgcattagcccattcttccatcagatttggtattggtagatttagtactgaagaggaggacgactacgtcgttaaggccg<br>tttctgacagagtaaaattcttgagggaactttcaccattatgggaaatggttcaagaaggtattgacttaaactccatcaaa<br>tggtcaggtcattgagtgtttttatttgttgtattttttttttttagagaaaatcctccaatatataaattaggaatcatagtttcat<br>gattttctgttacacctaactttttgtgtggtgccctcctccttgtcaatattaatgttaaagtgcaattctttttccttatcacgttg<br>agccattagtatcaatttgcttacctgtattcctttacatcctcctttttctccttcttgataaatgtatgtagattgcgtatatagttt<br>cgtctaccctatgaacatattccattttgtaatttcgtgtcgtttctattatgaatttcatttataaagtttatgtacaaatatcataa<br>aaaaagagaatctttttaagcaaggattttcttaacttcttcggcgacagcatcaccgacttcggtggtactgttggaacca<br>cctaaatcaccagttctgatacctgcatccaaaacctttttaactgcatcttcaatggccttaccttcttcaggcaagttcaat<br>gacaatttcaacatcattgcagcagacaagatagtggcgatagggttgaccttattctttggcaaatctggagcagaaccg<br>tggcatggttcgtacaaaccaaatgcggtgttcttgtctggcaaagaggccaaggacgcagatggcaacaaacccaag<br>gaacctgggataacggaggcttcatcggagatgatatcaccaaacatgttgctggtgattataataccatttaggtgggtt<br>gggttcttaactaggatcatggcggcagaatcaatcaattgatgttgaaccttcaatgtagggaattcgttcttgatggtttc<br>ctccacagtttttctccataatcttgaagaggccaaaacattagctttatccaaggaccaaataggcaatggtggctcatgtt<br>gtagggccatgaaagcggccattcttgtgattctttgcacttctggaacggtgtattgttcactatcccaagcgacaccatc<br>accatcgtcttcctttctcttaccaaagtaaatacctcccactaattctctgacaacaacgaagtcagtacctttagcaaattg<br>tggcttgattggagataagtctaaaagagagtcggatgcaaagttacatggtcttaagttggcgtacaattgaagttctttac<br>ggatttttagtaaaccttgttcaggtctaacactaccggtaccccatttaggaccacccacagcacctaacaaaacggcat<br>cagccttcttggaggcttccagcgcctcatctggaagtggaacacctgtagcatcgatagcagcaccaccaattaaatga<br>ttttcgaaatcgaacttgacattggaacgaacatcagaaatagctttaagaaccttaatggcttcggctgtgatttcttgacc<br>aacgtggtcacctggcaaaacgacgatcttcttaggggcagacattagaatggtatatccttgaaatatatatatatatattg<br>ctgaaatgtaaaaggtaagaaaagttagaaagtaagacgattgctaaccacctattggaaaaaacaataggtccttaaat<br>aatattgtcaacttcaagtattgtgatgcaagcatttagtcatgaacgcttctctattctatatgaaaagccggttccggcgct<br>ctcacctttcctttttctcccaatttttcagttgaaaaaggtatatgcgtcaggcgacctctgaaattaacaaaaaatttccagt<br>catcgaatttgattctgtgcgatagcgcccctgtgtgttctcgttatgttgaggaaaaaaataatggttgctaagagattcga<br>actcttgcatcttacgatacctgagtattcccacagttaactgcggtcaagatatttcttgaatcaggcgccttagaccgctc<br>ggccaaacaaccaattacttgttgagaaatagagtataattatcctataaatataacgtttttgaacacacatgaacaagga<br>agtacaggacaattgattttgaagagaatgtggattttgatgtaattgttgggattccatttttaataaggcaataatattaggt<br>atgtagatatactagaagttctcctcgaccggtcgatatgcggtgtgaaataccgcacagatgcgtaaggagaaaatacc<br>gcatcaggaaattgtaagcgttaatattttgttaaaattcgcgttaaatttttgttaaatcagctcattttttaaccaataggccg<br>aaatcggcaaaatcccttataaatcaaaagaatagaccgagatagggttgagtgttgttccagtttggaacaagagtcca<br>ctattaaagaacgtggactccaacgtcaaagggcgaaaaaccgtctatcagggcgatggcccactacgtgaaccatca<br>ccctaatcaagttttttggggtcgaggtgccgtaaagcactaaatcggaaccctaaagggagcccccgatttagagcttg<br>acggggaaagccggcgaacgtggcgagaaaggaagggaagaaagcgaaaggagcgggcgctagggcgctggc<br>aagtgtagcggtcacgctgcgcgtaaccaccacacccgccgcgcttaatgcgccgctacagggcgcgtccattcgcc<br>attcaggctgcgcaactgttgggaagggcgatcggtgcgggcctcttcgctattacgccaggaataaaaacacgctttt<br>tcagttcgagtttatcattatcaatactgccatttcaaagaatacgtaaataattaatagtagtgattttcctaactttatttagtc<br>aaaaaattagccttttaattctgctgtaacccgtacatgcccaaaataggggggcgggttacacagaatatataacatcgta<br>ggtgtctgggtgaacagtttattcctggcatccactaaatataatggagcccgctttttaagctggcatccagaaaaaaaa<br>gaatcccagcaccaaaatattgttttcttcaccaaccatcagttcataggtccattctcttagcgcaactacagagaacagg<br>ggcacaaacaggcaaaaaacgggcacaacctcaatggagtgatgcaacctgcctggagtaaatgatgacacaaggc<br>aattgacccacgcatgtatctatctcattttcttacaccttctattaccttctgctctctctgatttggaaaaagctgaaaaaaa<br>ggttgaaaccagttccctgaaattattcccctacttgactaataagtatataaagacggtaggtattgattgtaattctgtaaat<br>ctatttcttaaacttcttaaattctactttatagttagtcttttttttagttttaaaacaccaagaacttagtttcgaataaacacac

	ataaacaaacaaaatggcttcagaaaaagaaattaggagagagagattcttgaacgttttccctaaattagtagaggaatt<br>gaacgcatcgcttttggcttacggtatgcctaaggaagcatgtgactggtatgcccactcattgaactacaacactccagg<br>cggtaagctaaatagaggtttgtccgttgtggacacgtatgctattctctccaacaagaccgttgaacaattggggcaaga<br>agaatacgaaaaggttgccattctaggttggtgcattgagttgttgcaggcttactggttggtcgccgatgatatgatggac<br>aagtccattaccagaagaggccaaccatgttggtacaaggttcctgaagttggggaaattgccatctgggacgcattcat<br>gttagaggctgctatctacaagcttttgaaatctcacttcagaaacgaaaaatactacatagatatcaccgaattgttccatg<br>aggtcaccttccaaaccgaattgggccaattgatggacttaatcactgcacctgaagacaaagtcgacttgagtaagttct<br>ccctaaagaagcactccttcatagttactttcaagactgcttactattctttctacttgcctgtcgcattggccatgtacgttgc<br>cggtatcacggatgaaaaggatttgaaacaagccagagatgtcttgattccattgggtgaatacttccaaattcaagatga<br>ctacttagactgcttcggtaccccagaacagatcggtaagatcggtacagatatccaagataacaaatgttcttgggtaat<br>caacaaggcattggaacttgcttccgcagaacaaagaaagactttagacgaaaattacggtaagaaggactcagtcgca<br>gaagccaaatgcaaaaagattttcaatgacttgaaaattgaacagctataccacgaatatgaagagtctattgccaaggat<br>ttgaaggccaaaatttctcaggtcgatgagtctcgtggcttcaaagctgatgtcttaactgcgttcttgaacaaagtttacaa<br>gagaagcaaaatggaagcccatcaaattagaaggtctggtaattatcaaccagccttgtgggattccaactacattcaatc<br>tttgaacactccatacaccgaagaaagacacttggatagaaaagccgaattgatcgttcaagtcaggatcttgttgaaaga<br>aaagatggaaccagtccagcagttggaattgattcacgatttgaaatacttgggcctgtccgatttttttccaggacgaaatc<br>aaagaaatcttgggcgttatctacaacgaacataagtgcttccataacaacgaagttgagaagatggacttgtactttactg<br>ctttgggtttccgtttgttgagacaacatggtttcaacatctcccaagatgttttcaactgcttcaagaacgaaaagggtattg<br>acttcaaggcttctttggctcaagataccaaaggtatgttgcaattatacgaggccagtttcttgttgagaaagggtgaaga<br>tactttggaattggctagagaatttgctactaagtgcttgcagaaaaagttggatgaaggtggtaacgaaatcgacgaaaa<br>tttgttgttgtggatcagacactctttggacttgccattgcattggagaatccaatctgttgaagctagatggttcattgatgct<br>tatgctagaaggccagatatgaacccattgatttttgaattggccaagctgaacttcaacatcattcaagctactcaccagc<br>aagaattgaaggatttgtcaagatggtggtccagattgtgttttccagaaaagttgccattcgtcagagatagattggtcga<br>atctttttttgggccgttggtatgtttgaaccacatcaacacggttaccaaagaaaaatggctgctaccattatcgttttggc<br>caccgttattgatgacatctatgatgtttacggcaccttggatgaattggagttgtttactgataccttcaagagatgggatac<br>cgaatctattactagattgccctactacatgcaattgtgttattggggtgttcacaactacatctctgatgctgcttacgacatc<br>ttgaaagaacacggtttttctgcttgcagtacctgagaaaatccgttgttgatttggttgaagcctacttccatgaagctaag<br>tggtatcattctggttacactccatctttggacgagtatttgaacattgccaaaatctctgttgcttccccagctattatttctcc<br>aacttactttactttcgccaacgcttctcatgataccgctgttattgattccttgtaccaataccacgacattttgtgtttggccg<br>gtattatcttgagattgccagatgatttgggcacctcttactttgaattagcaagaggtgatgttccaaagaccattcagtgtt<br>acatgaaggaaactaacgcctctgaagaagaagctgttgaacacgttaagttcttgattagagaagcctggaaggatatg<br>aacactgctattgctgctggttatccatttccagatggtatggttgctggtgctgctaatattggtagagttgctcaattcatct<br>acttgcatggtgatggttttggtgtccaacattctaagacctacgaacatattgctggtttgttgttcgaaccatacgcttgag<br>tgaatttactttaaatcttgcatttaaataaattttctttttatagctttatgacttagtttcaatttatatactattttaatgacattttcg<br>attcattgattgaaagctttgtgtttttttcttgatgcgctattgcattgttcttgtctttttcgccacatgtaatatctgtagtagata<br>cctgatacattgtggatgctgagtgaaattttagttaataatggaggcgctcttaataattttggggatattggcttttttttttaa<br>agtttacaaatgaatttttttccgccaggatccctgcattaatgaatcggccaacgcgcggggagaggcggtttgcgtattg<br>ggcgctcttccgcttcctcgctcactgactcgctgcgctcggtcgttcggctgcggcgagcggtatcagctcactcaaag<br>gcggtaatacggttatccacagaatcaggggataacgcaggaaagaacatgtgagcaaaaggccagcaaaaggcca<br>ggaaccgtaaaaaggccgcgttgctggcgtttttccataggctccgcccccctgacgagcatcacaaaaatcgacgctc<br>aagtcagaggtggcgaaacccgacaggactataaagataccaggcgtttccccctggaagctccctcgtgcgctctcct<br>gttccgaccctgccgcttaccggatacctgtccgcctttctcccttcgggaagcgtggcgctttctcatagctcacgctgta<br>ggtatctcagttcggtgtaggtcgttcgctccaagctgggctgtgtgcacgaaccccccgttcagcccgaccgctgcgc<br>cttatccggtaactatcgtcttgagtccaacccggtaagacacgacttatcgccactggcagcagccactggtaacagga<br>ttagcagagcgaggtatgtaggcggtgctacagagttcttgaagtggtggcctaactacggctacactagaagaacagt<br>atttggtatctgcgctctgctgaagccagttaccttcggaaaaagagttggtagctcttgatccggcaaacaaaccaccgc<br>tggtagcggtggtttttttgtttgcaagcagcagattacgcgcagaaaaaaaggatctcaagaagatcctttgatcttttcta<br>cggggtctgacgctcagtggaacgaaaactcacgttaagggattttggtcatgagattatcaaaaaggatcttcacctag<br>atccttttaaattaaaaatgaagttttaaatcaatctaaagtatatatgagtaaacttggtctgacagttaccaatgcttaatca<br>gtgaggcacctatctcagcgatctgtctatttcgttcatccatagttgcctgactccccgtcgtgtagataactacgatacgg<br>gagggcttaccatctggccccagtgctgcaatgataccgcgagacccacgctcaccggctccagatttatcagcaataa<br>accagccagccggaagggccgagcgcagaagtggtcctgcaactttatccgcctccatccagtctattaattgttgccg<br>ggaagctagagtaagtagttcgccagttaatagtttgcgcaacgttgttgccattgctacaggcatcgtggtgtcacgctc<br>gtcgtttggtatggcttcattcagctccggttcccaacgatcaaggcgagttacatgatcccccatgttgtgcaaaaaagc

	ggttagctccttcggtcctccgatcgttgtcagaagtaagttggccgcagtgttatcactcatggttatggcagcactgcat aattctcttactgtcatgccatccgtaagatgcttttctgtgactggtgagtactcaaccaagtcattctgagaatagtgtatg cggcgaccgagttgctcttgcccggcgtcaatacgggataataccgcgccacatagcagaactttaaaagtgctcatcat tggaaaacgttcttcggggcgaaaactctcaaggatcttaccgctgttgagatccagttcgatgtaacccactcgtgcacc caactgatcttcagcatcttttactttcaccagcgtttctgggtgagcaaaaacaggaaggcaaaatgccgcaaaaaagg gaataagggcgacacggaaatgttgaatactcatactcttcctttttcaatattattgaagcatttatcagggttattgtctcat gagcggatacatatttgaatgtatttagaaaaataaacaaataggggttccgcgcacatttccccgaaaagtgccacctga cgtctaagaaaccattattatcatgacattaacctataaaaataggcgtatcacgaggccctttcgtc

**Name**	**pKZ069**
**Sequence**	tcgcgcgtttcggtgatgacggtgaaaacctctgacacatgcagctcccggagacggtcacagcttgtctgtaagcgga<br>tgccgggagcagacaagcccgtcagggcgcgtcagcgggtgttggcgggtgtcggggctggcttaactatgcggcat<br>cagagcagattgtactgagagtgcaccataaattcctattgtgagggtcagttatttcatccagatataacccgagaggaa<br>acttcttagcgtctgttttcgtaccataaggcagttcatgaggtatattttcgttattgaagcccagctcgtgaatgcttaatgc<br>tgctgaactggtgtccatgtcgcctagctacgcaatctccacaggctgcaaaggttttgtctcaagagcaatgttattgtgc<br>accccgtaattggtcaacaagtttaatctgtgcttgtccaccagctctgtcgtaaccttcagttcatcgactatctgaagaaat<br>ttactaggaatagtgccatggtacagcaaccgagaatggcaatttctactcgggttcagcaacgctgcataaacgctgttg<br>gtgccgtagacatattcgaagataggattatcattcataagtttcagagcaatgtccttattctggaacttggatttatggctct<br>tttggtttaatttcgcctgattcttgatctcctttagcttctcgacgtgggcctttttcttgccatatggatccgctgcacggtcct<br>gttccctagcatgtacgtgagcgtatttccttttaaaccacgacgctttgtcttcattcaacgtttcccattgtttttttctactatt<br>gctttgctgtgggaaaaacttatcgaaagatgacgactttttcttaattctcgttttaagagcttggtgagcgctaggagtca<br>ctgccaggtatcgtttgaacacggcattagtcagggaagtcataacacagtcctttcccgcaattttctttttctattactcttg<br>gcctcctctagtacactctatatttttttatgcctcggtaatgattttcattttttttttccacctagcggatgactctttttttttctta<br>gcgattggcattatcacataatgaattatacattatataaagtaatgtgatttcttcgaagaatatactaaaaaatgagcaggc<br>aagataaacgaaggcaaagatgacagagcagaaagccctagtaaagcgtattacaaatgaaaccaagattcagattgc<br>gatctctttaaagggtggtcccctagcgatagagcactcgatcttcccagaaaaagaggcagaagcagtagcagaaca<br>ggccacacaatcgcaagtgattaacgtccacacaggtatagggtttctggaccatatgatacatgctctggccaagcatt<br>ccggctggtcgctaatcgttgagtgcattggtgacttacacatagacgaccatcacaccactgaagactgcgggattgct<br>ctcggtcaagcttttaaagaggcgctaggggccgtgcgtggagtaaaaaggtttggatcaggatttgcgcctttggatga<br>ggcactttccagagcggtggtagatctttcgaacaggccgtacgcagttgtcgaacttggtttgcaaagggagaaagta<br>ggagatctctcttgcgagatgatcccgcattttcttgaaagctttgcagaggctagcagaattaccctccacgttgattgtct<br>gcgaggcaagaatgatcatcaccgtagtgagagtgcgttcaaggctcttgcggttgccataagagaagccacctcgcc<br>caatggtaccaacgatgttccctccaccaaaggtgttcttatgtagtgacaccgattatttaaagctgcagcatacgatatat<br>atacatgtgtatatatgtatacctatgaatgtcagtaagtatgtatacgaacagtatgatactgaagatgacaaggtaatgca<br>tcattctatacgtgtcattctgaacgaggcgcgctttccttttttctttttgctttttcttttttttttctcttgaactcgacggatctatg<br>cggtgtgaaataccgcacagatgcgtaaggagaaaataccgcatcaggaaattgtaagcgttaatattttgttaaaattcg<br>cgttaaatttttgttaaatcagctcattttttaaccaataggccgaaatcggcaaaatcccttataaatcaaaagaatagaccg<br>agatagggttgagtgttgttccagtttggaacaagagtccactattaaagaacgtggactccaacgtcaaagggcgaaaa<br>accgtctatcagggcgatggcccactacgtgaaccatcaccctaatcaagttttttggggtcgaggtgccgtaaagcact<br>aaatcggaaccctaaagggagcccccgatttagagcttgacggggaaagccggcgaacgtggcgagaaaggaagg<br>gaagaaagcgaaaggagcgggcgctagggcgctggcaagtgtagcggtcacgctgcgcgtaaccaccacacccgc<br>cgcgcttaatgcgccgctacagggcgcgtccattcgccattcaggctgcgcaactgttgggaagggcgatcggtgcgg<br>gcctcttcgctattacgccagctggcgaaagggggatgtgctgcaaggcgattaagttgggtaacgccagggttttccc<br>agtcacgacgttgtaaaacgacggccagtgagcgcgcgtaatacgactcactatagggcgaattgggtaccgggccc<br>cccctcgagttgtcctctgaggacataaaatacacaccgagattcatcaactcattgctggagttagcatatctacaattgg<br>gtgaaatggggagcgatttgcaggcatttgctcggcatgccggtagaggtgtggtcaataagagcgacctcatgctata<br>cctgagaaagcaacctgacctacaggaaagagttactcaagaataagaattttcgttttaaaacctaagagtcactttaaa<br>atttgtatacacttatttttttataacttatttaataataaaaatcataaatcataagaaattcgcggccggccgtttttagtttctg<br>taagacctaacggtgttgttaatggcggaaatggaatacttcaactcatcttccaaggtttggtcaatatcggtatccaaagt<br>ttcagcgaacatagcttcgtattcagcaatagacaattcggttctgttatccaacaaggccaagtgagtttctttttgcaagtg<br>gttttgataaccggcaaccaattcaccagtgaaaaattcagcaacggcacctgaaccataagaaaacaaaccaatttgat<br>taccggcggtcaaagtagtagcgttttccaacaaagagatcaggcccaagtataatgagccagtatacaagttaccgact<br>cttcttgagtagatgatggactcttcgtatctagccaagattctttcttgttcggcttcggtttgatcagaaatcttagccaaca<br>aagccttcttacccatcttagtgtatggaatatggaatgccaaagcatcgtaatcagcgaaatccaaaccagttctttttttgt<br>gttcatcccaaacttgggcgaaagattgaatgtaggtttcgttagacaatggaccatcaaccattggatatggatgaccag<br>ttggcctccaaaaatcgtagatgtcttgtgtcaacatgacgttatcttctttcaaggccaaaattctaggttcagaagcaacc<br>aacatagcaacagcaccagcaccttgagtaggttcaccaccagaatttaaaccgtatttggcaatatcagcagcaacaac<br>caaaaccttcttatctgggtgcaatgcaacgtgatttttagctaattgtaaaccagcagtagcgccgtaacaagcctctttga<br>tttcaaaggacctagcgaatggttgaatacccatcaatctatgcaagacaacagcagcagctttagattcatcaatggaag<br>attcggtgccaacgataaccatatcaatggcttctttgtcctcttttgtcaaaatggcttcagcagcattagcagcaaaagta<br>acgatgtcttgagaaattgggttaacagccatttgatcttgaccgataccaatatggaacttacctggatcaacatttctagc

	ttcagccaaagcagtcatgtcaatgtagtaaggtggaacgaaaaaagaaatcttgtcgatgccaatggtcataaaccctа<br>ggtgtatatgagatagttgattgtatgcttggtatagcttgaaatattgtgcagaaaaagaaacaaggaagaaagggaac<br>gagaacaatgacgaggaaacaaaagattaataattgcaggtctatttatacttgatagcaagacagcaaacttttttttatttc<br>aaattcaagtaactggaaggaaggccgtataccgttgctcattagagagtagtgtgcgtgaatgaaggaaggaaaaagt<br>ttcgtgtgcttcgagatacccctcatcagctctggaacaacgacatctgttggtgctgtctttgtcgttaatttttcctttagtg<br>tcttccatcatttttttgtcattgcggatatggtgagacaacaacgggggagagagaaaagaaaaaaaaagaaaagaagt<br>tgcatgcgcctattattacttcaatagatggcaaatggaaaaagggtagtgaaacttcgatatgatgatggctatcaagtct<br>agggctacagtattagttcgttatgtaccaccatcaatgaggcagtgtaattggtgtagtcttgtttagcccattatgtcttgtc<br>tggtatctgttctattgtatatctcccctccgccacctacatgttagggagaccaacgaaggtattataggaatcccgatgta<br>tgggtttggttgccagaaaagaggaagtccatattgtacacccccgcgggaataaaaacacgctttttcagttcgagttt<br>atcattatcaatactgccatttcaaagaatacgtaaataattaatagtagtgattttcctaactttatttagtcaaaaaattagcct<br>tttaattctgctgtaacccgtacatgcccaaaatagggggcgggttacacagaatatataacatcgtaggtgtctgggtga<br>acagtttattcctggcatccactaaatataatggagcccgctttttaagctggcatccagaaaaaaaaagaatcccagcac<br>caaaatattgttttcttcaccaaccatcagttcataggtccattctcttagcgcaactacagagaacaggggcacaaacag<br>gcaaaaaacgggcacaacctcaatggagtgatgcaacctgcctggagtaaatgatgacacaaggcaattgacccacg<br>catgtatctatctcattttcttacaccttctattaccttctgctctctctgatttggaaaaagctgaaaaaaaaaggttgaaacca<br>gttccctgaaattattcccctacttgactaataagtatataaagacggtaggtattgattgtaattctgtaaatctatttcttaaa<br>cttcttaaattctacttttatagttagtcttttttttagttttaaaacaccaagaacttagtttcgaataaacacacataaacaaaca<br>aagtcgaatgaagaccgttgttatcattgatgctttgagaactccaatcggtaagtacaaaggttccttgtctcaagtttctg<br>ctgttgatttgggtactcatgttactactcagttgttgaagagacactccaccatctctgaagaaattgatcaagtcattttcg<br>gcaacgttttacaagctggtaatggtcaaaatccagctagacaaattgccatcaattctggtttgtctcacgaaattccagct<br>atgactgttaacgaagtttgtggttctggtatgaaggctgttattttggctaaacagttgatccaattaggtgaagccgaagtt<br>ttgattgctggtggtattgaaaacatgtctcaagctccaaagttgcagagattcaactacgaaactgaatcttacgatgccc<br>cattctcttctatgatgtatgatggtttgaccgatgctttttctggtcaagctatgggtttgactgctgaaaatgttgctgaaaa<br>gtaccacgttaccagagaagaacaagatcaattctctgttcactctcagttgaaagctgctcaagctcaagctgaaggtat<br>tttgctgacgaaattgctccattggaagtctctggtactttggttgaaaaagacgaaggtatcagaccaaactcctctgttg<br>aaaaattgggcactttgaaaaccgtgttcaaagaagatggtactgttactgctggtaacgcttctactattaacgatggtgct<br>tccgctttgattattgcctctcaagaatatgctgaagctcatggtttgccatacttggccattattagagattctgtcgaagttg<br>gtattgacccagcttatatgggtatttctccaatcaaggccatccaaaagttgttggctagaaatcagttgaccactgaaga<br>gattgacttgtacgaaatcaatgaagcctttgctgctacctctattgtcgttcaaagagaattggctttgccagaagaaaag<br>gttaatatctatggtggtggtatctctttgggtcatgctattggtgctacaggtgctagattattgacttctttgtcctaccagct<br>gaaccagaaagaaaaaaagtatggtgttgcctccttgtgtataggtggtggtttgggtttagctatgttgttggaaagacca<br>caacagaagaagaactccagattctatcaaatgtccccagaggaaagattggcctctttgttgaatgaaggtcaaatttcc<br>gctgacaccaagaaagagtttgaaaacactgctttgtcctctcaaattgctaaccacatgatcgagaaccagatttctgaa<br>actgaagttccaatgggtgttggcttgcatttgacagttgacgaaacagattatttggtcccaatggctactgaagaaccat<br>ctgttattgctgcattgtctaacggtgctaaaattgctcaaggtttcaagaccgttaaccagcaaagattgatgagaggtca<br>gatcgtttttttacgatgttgcagatgccgaatccttgattgatgaattgcaagttagagaaaccgaaatcttccaacaagcc<br>gaattgtcttatccatctattgtcaaaagaggtggcggtttgagagacttgcagtatagagcttttgacgagtctttcgtttcc<br>gttgatttttttggttgatgtcaaggatgctatgggtgccaatatcgttaatgctatgttggaaggtgttgccgagttgtttagag<br>aatggtttgctgaacagaagatcctgttctccattttgtctaactacgctaccgaatctgttgttactatgaagactgctatccc<br>agtttccagattgtccaaaggttctaacggtagagaaatcgctgaaaagatagttttggcttccagatatgcctctttggacc<br>catacagagctgttactcataacaagggtattatgaacggtatcgaagcagttgttttggcaactggtaatgatacaagag<br>cagtttcagcttcttgtcatgcttttgctgtaaaagaaggtagataccaaggtttgacatcttggactttggatggtgaacaac<br>tgattggtgaaatctctgttccattggctttagctacagttggtggtgctactaaggttttgccaaaatctcaagctgctgccg<br>atttgttggctgttactgatgctaaagaattgtccagagttgttgctgctgttggtttagcacaaaacttggctgctttaagag<br>ctttggtttccgaaggtattcaaaaaggtcacatggcattgcaagctagatcattggctatgaccgttggtgccactggtaa<br>agaagttgaagctgttgctcaacagttgaagaggcaaaagactatgaatcaagatagagccttggccatcttgaacgattt<br>gagaaaacaatgaggccgcgtgaatttactttaaatcttgcatttaaataaattttctttttatagctttatgacttagtttcaattt<br>atatactattttaatgacattttcgattcattgattgaaagctttgtgtttttttcttgatgcgctattgcattgttcttgtctttttcgcc<br>acatgtaatatctgtagtagatacctgatacattgtggatgctgagtgaaattttagttaataatggaggcgctcttaataattt<br>tggggatattggcttttttttttaaagtttacaaatgaatttttttccgccaggatttaattaaccacacaccatagcttcaaaatgt<br>ttctactcctttttttactcttccagattttctcggactccgcgcatcgccgtaccacttcaaaacacccaagcacagcatacta<br>aatttcccctctttcttcctctagggtgtcgttaattacccgtactaaaggtttggaaaagaaaaaagagaccgcctcgtttct<br>ttttcttcgtcgaaaaaggcaataaaaatttttatcacgtttctttttcttgaaaattttttttttgatttttttctctttcgatgacctc

	ccattgatatttaagttaataaacggtcttcaatttctcaagtttcagtttcatttttcttgttctattacaactttttttacttcttgctc<br>attagaaagaaagcatagcaatctaatctaagttttaattacaaaatgtctatggcttcttgggttacaaaagttgctgctgaa<br>gaattggttgctccatgtgaacctactccatgtgctactttgccattgtcatctattgatcatgctttgggtttagccttcatggt<br>cgaaatgatttctatctacccaaacaacaacagacaacatcatcacccagctgctaaagttattcaagaagccttggctaa<br>agccctggttccatattatccagttgctggtagattggtttcttctggtggtgattgtgttgaagttgcttgtaatggtgaaggt<br>gtttggtttgttgaagctatggttactgatcactctttgaacggtttgaacgattgggaatctatcccaagatcagttgttaag<br>gatgaattgattccaggttgcccatctcatttgaagcaagaagaaatgatcatgatgatgcaagtcacccattttcaatgtg<br>gtggttttatcgttggcttgaagttcaaccatttggttttcgatggtctaggtttcggtcaattcttgaaagccattggtgaaatt<br>gcttgtggtagagcacatccatctgttgatccaatttggtacagagaagctattccagttccattcatgctgtccaagtctatt<br>tctttccccattaccaaaattgacatcgtcaactcctcttacgacttctctattcaaaccatcaagagattgaagggtcaaatt<br>gctaacgaaacctctaaccagttcactaccttcgaagttgttgttgctattttgtggaagtgtagaacccaagctattaacgc<br>tattggtgatgtttgtttgtctttcccagccaacgttagatatttgttggatcaattacctaaggccggtggttattatggtaact<br>gtatctacaacttgactgttaccgctacctctgaacaaatcaaaaaggcttcattggccgaattggtcagattgattagaaa<br>cgccaaagaatccttgcctaccaagtttaaagaatggacttccggtaatttcaaagaggacccttacaaaatctccagctc<br>ttacaactccttgatcttgtctgattggagatgcattgctattgatgaagctgattttggttggggtttgccacattttgtttcccc<br>aattatgcacgatattccatttgcctctggtatcattttgaagcagtctttgccaaaaggtggtgttcatttcgaaggtttggct<br>actatgaaggaacatgagaagagattcatcgacgagatcaacaagtttttggctaacgtcatgctgtgagcgaatttcttat<br>gatttatgatttttattattaaataagttataaaaaaaataagtgtatacaaattttaaagtgactcttaggttttaaaacgaaaat<br>tcttattcttgagtaactctttcctgtaggtcaggttgctttctcaggtatagcatgaggtcgctcttattgaccacacctctacc<br>ggcatgccgagcaaatgcctgcaaatcgctccccatttcacccaattgtagatatgctaactccagcaatgagttgatgaa<br>tctcggtgtgtattttatgtcctcagaggacaagagctccagcttttgttccctttagtgagggttaattgcgcgcttggcgta<br>atcatggtcatagctgtttcctgtgtgaaattgttatccgctcacaattccacacaacatacgagccggaagcataaagtgt<br>aaagcctggggtgcctaatgagtgagctaactcacattaattgcgttgcgctcactgcccgctttccagtcgggaaacct<br>gtcgtgccagctgcattaatgaatcggccaacgcgcggggagaggcggtttgcgtattgggcgctcttccgcttcctcg<br>ctcactgactcgctgcgctcggtcgttcggctgcggcgagcggtatcagctcactcaaaggcggtaatacggttatcca<br>cagaatcaggggataacgcaggaaagaacatgtgagcaaaaggccagcaaaaggccaggaaccgtaaaaaggccg<br>cgttgctggcgtttttccataggctccgcccccctgacgagcatcacaaaaatcgacgctcaagtcagaggtggcgaaa<br>cccgacaggactataaagataccaggcgtttccccctggaagctccctcgtgcgctctcctgttccgaccctgccgctta<br>ccggatacctgtccgcctttctcccttcgggaagcgtggcgctttctcatagctcacgctgtaggtatctcagttcggtgta<br>ggtcgttcgctccaagctgggctgtgtgcacgaaccccccgttcagcccgaccgctgcgccttatccggtaactatcgtc<br>ttgagtccaacccggtaagacacgacttatcgccactggcagcagccactggtaacaggattagcagagcgaggtatg<br>taggcggtgctacagagttcttgaagtggtggcctaactacggctacactagaagaacagtatttggtatctgcgctctgc<br>tgaagccagttaccttcggaaaaagagttggtagctcttgatccggcaaacaaaccaccgctggtagcggtggtttttttg<br>tttgcaagcagcagattacgcgcagaaaaaaaggatctcaagaagatcctttgatcttttctacggggtctgacgctcagt<br>ggaacgaaaactcacgttaagggattttggtcatgagattatcaaaaaggatcttcacctagatccttttaaattaaaaatga<br>agttttaaatcaatctaaagtatatatgagtaaacttggtctgacagttaccaatgcttaatcagtgaggcacctatctcagc<br>gatctgtctatttcgttcatccatagttgcctgactccccgtcgtgtagataactacgatacgggagggcttaccatctggcc<br>ccagtgctgcaatgataccgcgagacccacgctcaccggctccagatttatcagcaataaaccagccagccggaagg<br>gccgagcgcagaagtggtcctgcaactttatccgcctccatccagtctattaattgttgccgggaagctagagtaagtagt<br>tcgccagttaatagtttgcgcaacgttgttgccattgctacaggcatcgtggtgtcacgctcgtcgtttggtatggcttcattc<br>agctccggttcccaacgatcaaggcgagttacatgatcccccatgttgtgcaaaaaagcggttagctccttcggtcctcc<br>gatcgttgtcagaagtaagttggccgcagtgttatcactcatggttatggcagcactgcataattctcttactgtcatgccat<br>ccgtaagatgcttttctgtgactggtgagtactcaaccaagtcattctgagaatagtgtatgcggcgaccgagttgctcttg<br>cccggcgtcaatacgggataataccgcgccacatagcagaactttaaaagtgctcatcattggaaaacgttcttcggggc<br>gaaaactctcaaggatcttaccgctgttgagatccagttcgatgtaacccactcgtgcacccaactgatcttcagcatctttt<br>actttcaccagcgtttctgggtgagcaaaaacaggaaggcaaaatgccgcaaaaaagggaataagggcgacacggaa<br>atgttgaatactcatactcttcctttttcaatattattgaagcatttatcagggttattgtctcatgagcggatacatatttgaatgt<br>atttagaaaaataaacaaataggggttccgcgcacatttccccgaaaagtgccacctgacgtctaagaaaccattattatc<br>atgacattaacctataaaaataggcgtatcacgaggccctttcgtc

**Name**	**pKZ071**
**Sequence**	tcgcgcgtttcggtgatgacggtgaaaacctctgacacatgcagctcccggagacggtcacagcttgtctgtaagcgga<br>tgccgggagcagacaagcccgtcagggcgcgtcagcgggtgttggcgggtgtcggggctggcttaactatgcggcat<br>cagagcagattgtactgagagtgcaccataaattcctattgtgagggtcagttatttcatccagatataacccgagaggaa<br>acttcttagcgtctgttttcgtaccataaggcagttcatgaggtatattttcgttattgaagcccagctcgtgaatgcttaatgc<br>tgctgaactggtgtccatgtcgcctagctacgcaatctccacaggctgcaaaggttttgtctcaagagcaatgttattgtgc<br>accccgtaattggtcaacaagtttaatctgtgcttgtccaccagctctgtcgtaaccttcagttcatcgactatctgaagaaat<br>ttactaggaatagtgccatggtacagcaaccgagaatggcaatttctactcgggttcagcaacgctgcataaacgctgttg<br>gtgccgtagacatattcgaagataggattatcattcataagtttcagagcaatgtccttattctggaacttggatttatggctct<br>tttggtttaatttcgcctgattcttgatctcctttagcttctcgacgtgggcctttttcttgccatatggatccgctgcacggtcct<br>gttccctagcatgtacgtgagcgtatttccttttaaaccacgacgctttgtcttcattcaacgtttcccattgtttttttctactatt<br>gctttgctgtgggaaaaacttatcgaaagatgacgactttttcttaattctcgttttaagagcttggtgagcgctaggagtca<br>ctgccaggtatcgtttgaacacggcattagtcagggaagtcataacacagtcctttcccgcaattttctttttctattactcttg<br>gcctcctctagtacactctatattttttatgcctcggtaatgattttcattttttttttccacctagcggatgactcttttttttttctta<br>gcgattggcattatcacataatgaattatacattatataaagtaatgtgatttcttcgaagaatatactaaaaaatgagcaggc<br>aagataaacgaaggcaaagatgacagagcagaaagccctagtaaagcgtattacaaatgaaaccaagattcagattgc<br>gatctctttaaagggtggtcccctagcgatagagcactcgatcttcccagaaaaagaggcagaagcagtagcagaaca<br>ggccacacaatcgcaagtgattaacgtccacacaggtatagggtttctggaccatatgatacatgctctggccaagcatt<br>ccggctggtcgctaatcgttgagtgcattggtgacttacacatagacgaccatcacaccactgaagactgcgggattgct<br>ctcggtcaagcttttaaagaggcgctaggggccgtgcgtggagtaaaaaggtttggatcaggatttgcgcctttggatga<br>ggcactttccagagcggtggtagatctttcgaacaggccgtacgcagttgtcgaacttggtttgcaaagggagaaagta<br>ggagatctctcttgcgagatgatcccgcattttcttgaaagctttgcagaggctagcagaattaccctccacgttgattgtct<br>gcgaggcaagaatgatcatcaccgtagtgagagtgcgttcaaggctcttgcggttgccataagagaagccacctcgcc<br>caatggtaccaacgatgttccctccaccaaaggtgttcttatgtagtgacaccgattatttaaagctgcagcatacgatatat<br>atacatgtgtatatatgtatacctatgaatgtcagtaagtatgtatacgaacagtatgatactgaagatgacaaggtaatgca<br>tcattctatacgtgtcattctgaacgaggcgcgctttccttttttctttttgctttttcttttttttttctcttgaactcgacggatctatg<br>cggtgtgaaataccgcacagatgcgtaaggagaaaataccgcatcaggaaattgtaagcgttaatattttgttaaaattcg<br>cgttaaatttttgttaaatcagctcattttttaaccaataggccgaaatcggcaaaatcccttataaatcaaaagaatagaccg<br>agatagggttgagtgttgttccagtttggaacaagagtccactattaaagaacgtggactccaacgtcaaagggcgaaaa<br>accgtctatcagggcgatggcccactacgtgaaccatcaccctaatcaagttttttggggtcgaggtgccgtaaagcact<br>aaatcggaaccctaaagggagcccccgatttagagcttgacggggaaagccggcgaacgtggcgagaaaggaagg<br>gaagaaagcgaaaggagcgggcgctagggcgctggcaagtgtagcggtcacgctgcgcgtaaccaccacacccgc<br>cgcgcttaatgcgccgctacagggcgcgtccattcgccattcaggctgcgcaactgttgggaagggcgatcggtgcgg<br>gcctcttcgctattacgccagctggcgaaagggggatgtgctgcaaggcgattaagttgggtaacgccagggttttccc<br>agtcacgacgttgtaaaacgacggccagtgagcgcgcgtaatacgactcactatagggcgaattgggtaccgggccc<br>cccctcgagttgtcctctgaggacataaaatacacaccgagattcatcaactcattgctggagttagcatatctacaattgg<br>gtgaaatggggagcgatttgcaggcatttgctcggcatgccggtagaggtgtggtcaataagagcgacctcatgctata<br>cctgagaaagcaacctgacctacaggaaagagttactcaagaataagaattttcgttttaaaacctaagagtcactttaaa<br>atttgtatacacttattttttttataacttatttaataataaaaatcataaatcataagaaattcgcggccggccgtttttagtttctg<br>taagacctaacggtgttgttaatggcggaaatggaatacttcaactcatcttccaaggtttggtcaatatcggtatccaaagt<br>ttcagcgaacatagcttcgtattcagcaatagacaattcggttctgttatccaacaaggccaagtgagtttctttttgcaagtg<br>gttttgataaccggcaaccaattcaccagtgaaaaattcagcaacggcacctgaaccataagaaaacaaaccaatttgat<br>taccggcggtcaaagtagtagcgttttccaacaaagagatcaggcccaagtataatgagccagtatacaagttaccgact<br>cttcttgagtagatgatggactcttcgtatctagccaagattctttcttgttcggcttcggtttgatcagaaatcttagccaaca<br>aagccttcttacccatcttagtgtatggaatatggaatgccaaagcatcgtaatcagcgaaatccaaaccagttcttttttttgt<br>gttcatcccaaacttgggcgaaagattgaatgtaggtttcgttagacaatggaccatcaaccattggatatggatgaccag<br>ttggcctccaaaaatcgtagatgtcttgtgtcaacatgacgttatcttctttcaaggccaaaattctaggttcagaagcaacc<br>aacatagcaacagcaccagcaccttgagtaggttcaccaccagaatttaaaccgtatttggcaatatcagcagcaacaac<br>caaaaccttcttatctgggtgcaatgcaacgtgatttttagctaattgtaaaccagcagtagcgccgtaacaagcctctttga<br>tttcaaaggacctagcgaatggttgaatacccatcaatctatgcaagacaacagcagcagctttagattcatcaatggaag<br>attcggtgccaacgataaccatatcaatggcttctttgtcctcttttgtcaaaatggcttcagcagcattagcagcaaaagta<br>acgatgtcttgagaaattgggttaacagccatttgatcttgaccgataccaatatggaacttacctggatcaacatttctagc<br>ttcagccaaagcagtcatgtcaatgtagtaaggtggaacgaaaaaagaaatcttgtcgatgccaatggtcataaacccta<br>ggtgtatatgagatagttgattgtatgcttggtatagcttgaaatattgtgcagaaaaagaaacaaggaagaaagggaac

	gagaacaatgacgaggaaacaaaagattaataattgcaggtctatttatacttgatagcaagacagcaaacttttttttatttc<br>aaattcaagtaactggaaggaaggccgtataccgttgctcattagagagtagtgtgcgtgaatgaaggaaggaaaaagt<br>ttcgtgtgcttcgagatacccctcatcagctctggaacaacgacatctgttggtgctgtctttgtcgttaatttttttcctttagtg<br>tcttccatcatttttttgtcattgcggatatggtgagacaacaacgggggagagagaaaagaaaaaaaaagaaaagaagt<br>tgcatgcgcctattattacttcaatagatggcaaatggaaaaagggtagtgaaacttcgatatgatgatggctatcaagtct<br>agggctacagtattagttcgttatgtaccaccatcaatgaggcagtgtaattggtgtagtcttgtttagcccattatgtcttgtc<br>tggtatctgttctattgtatatctcccctccgccacctacatgttagggagaccaacgaaggtattataggaatcccgatgta<br>tgggtttggttgccagaaaagaggaagtccatattgtacacccccgcgggaataaaaaacacgctttttcagttcgagttt<br>atcattatcaatactgccatttcaaagaatacgtaaataattaatagtagtgattttcctaactttatttagtcaaaaaattagcct<br>tttaattctgctgtaacccgtacatgcccaaaataggggggcgggttacacagaatatataacatcgtaggtgtctgggtga<br>acagtttattcctggcatccactaaatataatggagcccgctttttaagctggcatccagaaaaaaaaagaatcccagcac<br>caaaatattgttttcttcaccaaccatcagttcataggtccattctcttagcgcaactacagagaacaggggcacaaacag<br>gcaaaaaacgggcacaacctcaatggagtgatgcaacctgcctggagtaaatgatgacacaaggcaattgacccacg<br>catgtatctatctcattttcttacaccttctattaccttctgctctctctgatttggaaaaagctgaaaaaaaaggttgaaacca<br>gttccctgaaattattcccctacttgactaataagtatataaagacggtaggtattgattgtaattctgtaaatctatttcttaaa<br>cttcttaaattctacttttatagttagtcttttttttagttttaaaacaccaagaacttagtttcgaataaacacacataaacaaaca<br>aagtcgaatgaagaccgttgttatcattgatgctttgagaactccaatcggtaagtacaaaggttccttgtctcaagtttctg<br>ctgttgatttgggtactcatgttactactcagttgttgaagagacactccaccatctctgaagaaattgatcaagtcattttcg<br>gcaacgttttacaagctggtaatggtcaaaatccagctagacaaattgccatcaattctggtttgtctcacgaaattccagct<br>atgactgttaacgaagtttgtggttctggtatgaaggctgttattttggctaaacagttgatccaattaggtgaagccgaagtt<br>ttgattgctggtggtattgaaaacatgtctcaagctccaaagttgcagagattcaactacgaaactgaatcttacgatgccc<br>cattctcttctatgatgtatgatggtttgaccgatgctttttctggtcaagctatgggtttgactgctgaaaatgttgctgaaaa<br>gtaccacgttaccagagaagaacaagatcaattctctgttcactctcagttgaaagctgctcaagctcaagctgaaggtat<br>tttgctgacgaaattgctccattggaagtctctggtactttggttgaaaaagacgaaggtatcagaccaaactcctctgttg<br>aaaaattgggcactttgaaaaccgtgttcaaagaagatggtactgttactgctggtaacgcttctactattaacgatggtgct<br>tccgctttgattattgcctctcaagaatatgctgaagctcatggtttgccatacttggccattattagagattctgtcgaagttg<br>gtattgacccagcttatatgggtatttctccaatcaaggccatccaaaagttgttggctagaaatcagttgaccactgaaga<br>gattgacttgtacgaaatcaatgaagcctttgctgctacctctattgtcgttcaaagagaattggctttgccagaagaaaag<br>gttaatatctatggtggtggtatctctttgggtcatgctattggtgctacaggtgctagattattgacttctttgtcctaccagct<br>gaaccagaaagaaaaaaagtatggtgttgcctccttgtgtataggtggtggtttgggtttagctatgttgttggaaagacca<br>caacagaagaagaactccagattctatcaaatgtccccagaggaaagattggcctctttgttgaatgaaggtcaaatttcc<br>gctgacaccaagaaagagtttgaaaacactgctttgtcctctcaaattgctaaccacatgatcgagaaccagatttctgaa<br>actgaagttccaatgggtgttggcttgcatttgacagttgacgaaacagattatttggtcccaatggctactgaagaaccat<br>ctgttattgctgcattgtctaacggtgctaaaattgctcaaggtttcaagaccgttaaccagcaaagattgatgagaggtca<br>gatcgtttttttacgatgttgcagatgccgaatccttgattgatgaattgcaagttagagaaaccgaaatcttccaacaagcc<br>gaattgtcttatccatctattgtcaaaagaggtggcggtttgagagacttgcagtatagagcttttgacgagtctttcgtttcc<br>gttgatttttttggttgatgtcaaggatgctatgggtgccaatatcgttaatgctatgttggaaggtgttgccgagttgtttagag<br>aatggtttgctgaacagaagatcctgttctccattttgtctaactacgctaccgaatctgttgttactatgaagactgctatccc<br>agtttccagattgtccaaaggttctaacggtagagaaatcgctgaaaagatagttttggcttccagatatgcctctttggacc<br>catacagagctgttactcataacaagggtattatgaacggtatcgaagcagttgttttggcaactggtaatgatacaagag<br>cagtttcagcttcttgtcatgcttttgctgtaaaagaaggtagataccaaggtttgacatcttggactttggatggtgaacaac<br>tgattggtgaaatctctgttccattggctttagctacagttggtggtgctactaaggttttgccaaaatctcaagctgctgccg<br>atttgttggctgttactgatgctaaagaattgtccagagttgttgctgctgttggtttagcacaaaacttggctgctttaagag<br>ctttggtttccgaaggtattcaaaaaggtcacatggcattgcaagctagatcattggctatgaccgttggtgccactggtaa<br>agaagttgaagctgttgctcaacagttgaagaggcaaaagactatgaatcaagatagagccttggccatcttgaacgattt<br>gagaaaacaatgaggccgcgtgaatttactttaaatcttgcatttaaataaattttctttttatagctttatgacttagtttcaattt<br>atatactattttaatgacattttcgattcattgattgaaagctttgtgtttttttcttgatgcgctattgcattgttcttgtctttttcgcc<br>acatgtaatatctgtagtagatacctgatacattgtggatgctgagtgaaattttagttaataatggaggcgctcttaataattt<br>tggggatattggcttttttttttaaagtttacaaatgaattttttccgccaggatttaattaaccacacaccatagcttcaaaatgt<br>ttctactcctttttttactcttccagattttctcggactccgcgcatcgccgtaccacttcaaaacacccaagcacagcatacta<br>aatttcccctctttcttcctctagggtgtcgttaattacccgtactaaaggtttggaaaagaaaaaagagaccgcctcgtttct<br>ttttcttcgtcgaaaaaggcaataaaaatttttatcacgtttctttttcttgaaaattttttttttgatttttttctctttcgatgacctc<br>ccattgatatttaagttaataaacggtcttcaatttctcaagtttcagtttcatttttcttgttctattacaactttttttacttcttgctc<br>attagaaagaaagcatagcaatctaatctaagttttaattacaaaatgaagttgttggaaggtaagagaatcatcgttactg

	gtggtgctcaaggtattggtgcttctgttgttagagcttatattgctgctggtgctacagttgcttctatggatatgaatgatac<br>cttgggtcaacaggttgtttctgaagctggtaaagctaatccaggttgtaaatccagatattaccattgcaacattgccgata<br>gaccagaagttgaaaaggcttttgctactgctgctgaagatatgggtggtttggatgttatggttaatgttgctggtgttcata<br>gacattctccaccagatgctattgccgaagaattatacgatatgttgttcagggttaatgtcttgggtactattaacactaacg<br>ctgttgcttacagattgatgaaaggtcaaggtatcggtaacatcatcaacttcggttctgaatctggtttgactggtgaaatc<br>aacaatgccttgtattctgctacaaaggctgctgttcatacttggactagaaatgttgctagacaatggggtccagatggta<br>ttagaatcaatgctgttttgccttacatggtcaccccaatgtatgttgattttagaaacgccttgtcctccgaagatttggctgc<br>tcatgatgctgctactaagactgatattccattaggtggtaagtttggtgatgccgataaggatttggctccagttatggttttt<br>ttggcttctgatgcttcccatttcatgactggtcaaatgtttccagttgatggtggtttgattgccgttagatagcgcgccgaa<br>ataaattgaattgaattgaaatcgatagatcaatttttttcttttctctttccccatcctttacgctaaaataatagtttattttattttt<br>gaatatttttatttatatacgtatatatagactattatttatcttttaatgattattaagatttttattaaaaaaaaattcgctcctcttt<br>taatgcctttatgcagtttttttttcccattcgatatttctatgttcgggttcagcgtattttaagtttaataactcgaaaattctgcg<br>ttcgttaaagctgagctccagcttttgttccctttagtgagggttaattgcgcgcttggcgtaatcatggtcatagctgtttcct<br>gtgtgaaattgttatccgctcacaattccacacaacatacgagccggaagcataaagtgtaaagcctggggtgcctaatg<br>agtgagctaactcacattaattgcgttgcgctcactgcccgctttccagtcgggaaacctgtcgtgccagctgcattaatg<br>aatcggccaacgcgcggggagaggcggtttgcgtattgggcgctcttccgcttcctcgctcactgactcgctgcgctcg<br>gtcgttcggctgcggcgagcggtatcagctcactcaaaggcggtaatacggttatccacagaatcaggggataacgca<br>ggaaagaacatgtgagcaaaaggccagcaaaaggccaggaaccgtaaaaaggccgcgttgctggcgtttttccatag<br>gctccgcccccctgacgagcatcacaaaaatcgacgctcaagtcagaggtggcgaaacccgacaggactataaagat<br>accaggcgtttccccctggaagctccctcgtgcgctctcctgttccgaccctgccgcttaccggatacctgtccgcctttct<br>cccttcgggaagcgtggcgctttctcatagctcacgctgtaggtatctcagttcggtgtaggtcgttcgctccaagctggg<br>ctgtgtgcacgaaccccccgttcagcccgaccgctgcgccttatccggtaactatcgtcttgagtccaacccggtaagac<br>acgacttatcgccactggcagcagccactggtaacaggattagcagagcgaggtatgtaggcggtgctacagagttctt<br>gaagtggtggcctaactacggctacactagaagaacagtatttggtatctgcgctctgctgaagccagttaccttcggaa<br>aaagagttggtagctcttgatccggcaaacaaaccaccgctggtagcggtggtttttttgtttgcaagcagcagattacgc<br>gcagaaaaaaaggatctcaagaagatcctttgatcttttctacggggtctgacgctcagtggaacgaaaactcacgttaa<br>gggattttggtcatgagattatcaaaaaggatcttcacctagatccttttaaattaaaaatgaagttttaaatcaatctaaagta<br>tatatgagtaaacttggtctgacagttaccaatgcttaatcagtgaggcacctatctcagcgatctgtctatttcgttcatccat<br>agttgcctgactccccgtcgtgtagataactacgatacgggagggcttaccatctggccccagtgctgcaatgataccgc<br>gagacccacgctcaccggctccagatttatcagcaataaaccagccagccggaagggccgagcgcagaagtggtcct<br>gcaactttatccgcctccatccagtctattaattgttgccgggaagctagagtaagtagttcgccagttaatagtttgcgcaa<br>cgttgttgccattgctacaggcatcgtggtgtcacgctcgtcgtttggtatggcttcattcagctccggttcccaacgatcaa<br>ggcgagttacatgatcccccatgttgtgcaaaaaagcggttagctccttcggtcctccgatcgttgtcagaagtaagttgg<br>ccgcagtgttatcactcatggttatggcagcactgcataattctcttactgtcatgccatccgtaagatgcttttctgtgactg<br>gtgagtactcaaccaagtcattctgagaatagtgtatgcggcgaccgagttgctcttgcccggcgtcaatacgggataat<br>accgcgccacatagcagaactttaaaagtgctcatcattggaaaacgttcttcggggcgaaaactctcaaggatcttacc<br>gctgttgagatccagttcgatgtaacccactcgtgcacccaactgatcttcagcatcttttactttcaccagcgtttctgggtg<br>agcaaaaacaggaaggcaaaatgccgcaaaaaagggaataagggcgacacggaaatgttgaatactcatactcttcct<br>ttttcaatattattgaagcatttatcagggttattgtctcatgagcggatacatatttgaatgtatttagaaaaataaacaaatag<br>gggttccgcgcacatttccccgaaaagtgccacctgacgtctaagaaaccattattatcatgacattaacctataaaaata<br>ggcgtatcacgaggccctttcgtc
